# Co-production in health policy and management: a comprehensive bibliometric review

**DOI:** 10.1186/s12913-020-05241-2

**Published:** 2020-06-05

**Authors:** Floriana Fusco, Marta Marsilio, Chiara Guglielmetti

**Affiliations:** Department of Economics, Management and Quantitative Methods (DEMM), Università degli Studi di Milano, via Conservatorio, 7, 20122 Milan, Italy

**Keywords:** Co-production, Health, Co-creation, Patient engagement, Bibliometric analysis, Co-citation analysis, Co-word analysis, Science mapping

## Abstract

**Background:**

Due to an increasingly elderly population, a higher incidence of chronic diseases and higher expectations regarding public service provision, healthcare services are under increasing strain to cut costs while maintaining quality. The importance of promoting systems of co-produced health between stakeholders has gained considerable traction both in the literature and in public sector policy debates. This study provides a comprehensive map of the extant literature and identifies the main themes and future research needs.

**Methods:**

A quantitative bibliometric analysis was carried out consisting of a performance analysis, science mapping, and a scientific collaboration analysis. Web of Science (WoS) was chosen to extract the dataset; the search was refined by language, i.e. English, and type of publication, i.e. journal academic articles and reviews. No time limitation was selected.

**Results:**

The dataset is made up of 295 papers ranging from 1994 to May 2019. The analysis highlighted an annual percentage growth rate in the topic of co-production of about 25%. The articles retrieved are split between 1225 authors and 148 sources. This fragmentation was confirmed by the collaboration analysis, which revealed very few long-lasting collaborations. The scientific production is geographically polarised within the EU and Anglo-Saxon countries, with the United Kingdom playing a central role. The intellectual structure consists of three main areas: public administration and management, service management and knowledge translation literature. The co-word analysis confirms the relatively low scientific maturity of co-production applied to health services. It shows few well-developed and central terms, which refer to traditional areas of co-production (e.g. public health, social care), and some emerging themes related to social and health phenomena (e.g. the elderly and chronic diseases), the use of technologies, and the recent patient-centred approach to care (patient involvement/engagement).

**Conclusions:**

The field is still far from being mature. Empirical practices, especially regarding co-delivery and co-management as well as the evaluation of their real impacts on providers and on patients are lacking and should be more widely investigated.

## Background

Co-production in public services has become the leitmotif of public policy reform [1–3]. It is considered a potential solution to the current and future challenges in the public sector, given that the expected benefits concern the improvement of the services provided, a greater economic and financial sustainability of the system, the more efficient use of resources, and the possibility of increasing the satisfaction level of citizens [4, 5].

Despite this growing interest, co-production is still a nebulous concept, a “*woolly-word*” in the field of public policy [[6]:640], a “*quite heterogeneous umbrella concept”* [[7]:1094], with a wide range of definitions [8]. In 1996, Ostrom [[9]:1073] defined it as “*the processes through which inputs, used to provide a good or a service, are contributed by individuals who are not in the same organization*”. This definition was subsequently interpreted in different ways, considering only the involvement of the users [6] or of any individual or entity external to the organisation [10]. There is some agreement that it covers the practices in which state actors (i.e. government agents serving in a professional capacity) and lay actors (i.e. members of the public, serving voluntarily as citizens or users) work together in any phase of the public service cycle (i.e. commissioning, design, delivery, and assessment) [11, 12]. Co-production implies that citizens are not merely recipients of services, but can act as co-producers in the design and the delivery of public services [3].

Of the various contexts of application, the literature seems to agree that the health sector is a reference point where the concepts of co-production can be analysed and put into practice [5, 13–18]. It is a sector where resources are being reduced and where there are increasing concerns about long-term economic sustainability. On the other hand, there are expectations of higher quality as well as a growing demand caused above all by an aging population and the rise in chronic diseases. This is forcing healthcare policymakers and managers to reduce healthcare costs (e.g. by reducing the length of hospital stays and readmission rates), and at the same time, improve the quality of the service and, more generally, the patients’ quality of life [19]. Patient engagement has become imperative in delivering high quality healthcare services [20]. This more active role of patients has transformed public service production: patients are asked to participate actively and act as consumer producers, next to and in interaction with healthcare professionals and other decision-makers in healthcare, such as policy makers [21, 22].

The pressure to create co-produced health services is increasing, and the debate is wide open on the nature of co-production, on how healthcare practices change in order to manage effective partnerships between clients and professionals and on the impacts of trying to optimise health outcomes. Despite the growing interest and the consequent increase in the number of publications on co-production in the healthcare sector [16], there is a lack of studies that provide a comprehensive picture of the structure and the development of this field.

Some co-production qualitative literature reviews have already been carried out. Only one specifically targeted the healthcare sector [16]; others have investigated the broader context of public services [5, 7]. All of them have focused on specific research questions (such as the aims, drivers/barriers and outcomes), but do not provide an overall and comprehensive picture of the healthcare co-production research field.

This paper aims to fill this gap with a quantitative bibliometric analysis on co-production in the healthcare sector. A quantitative review enhances the understanding of this research field and further informs the scientific debate on this topic. Specifically, using the main procedures of the bibliometric method (performance analysis, scientific collaboration analysis and science mapping), the work aims to *i)* quantify the research field and describe its main outputs and evolution; *ii)* analyse the collaboration practices and map the social structure of the field; *iii)* define the intellectual structure and understand the main conceptualizations and theoretical approaches; *iv)* identify the most investigated themes and propose future avenues for research.

## Methods

Bibliometric analysis has been increasingly used both in social sciences [e.g. [23–26]]; and with specific reference to the health field [e.g. [27–29]], and medical science [e.g. [30, 31]]. It is based on the statistical measurement of science, scientists, or scientific activity and it is, therefore, considered a more objective and reproducible method with which to develop a review process compared to other techniques [32, 33].

The process of data collection and data analysis is detailed below.

### Data collection

Data were retrieved from the Web of Science (WoS), and specifically from the Science Citation Index Expanded (SCI expanded) and the Social Science Citation Index (SSCI) [34, 35]. The search criteria were “co-production AND health* OR coproduction AND health*” in the string “topic” (that is title, abstract and keywords). The search did not include correlated words, such as *engagement*, *involvement* and *co-creation*, given that these are different concepts from *co-production*, although strictly linked to it [6]. The query was launched on 10 May 2019 and resulted in the retrieval of 555 documents.

The search was then refined by language (English) and document type (article and review) [36]. Although co-production first appeared in the 1970s, it has begun to receive particular attention in recent years. No limitation time was selected in order to gather the evolution over time since its first appearance in literature. The filtering stage produced 500 documents.

To avoid including papers that were not related to the topic, i.e. not containing the concept of co-production (as defined above), and/or not referring to the health field, the collection was screened in terms of titles and abstracts. The excluded papers focused on microbiology, biochemistry, pharmacology, environmental science and ecology. This phase was first carried out by one author, and then checked by two other authors and any discrepancies in the evaluation were discussed. This screening thus reduced the risk of including unrelated articles or, conversely, excluding pertinent ones.

**Fig. 1:**
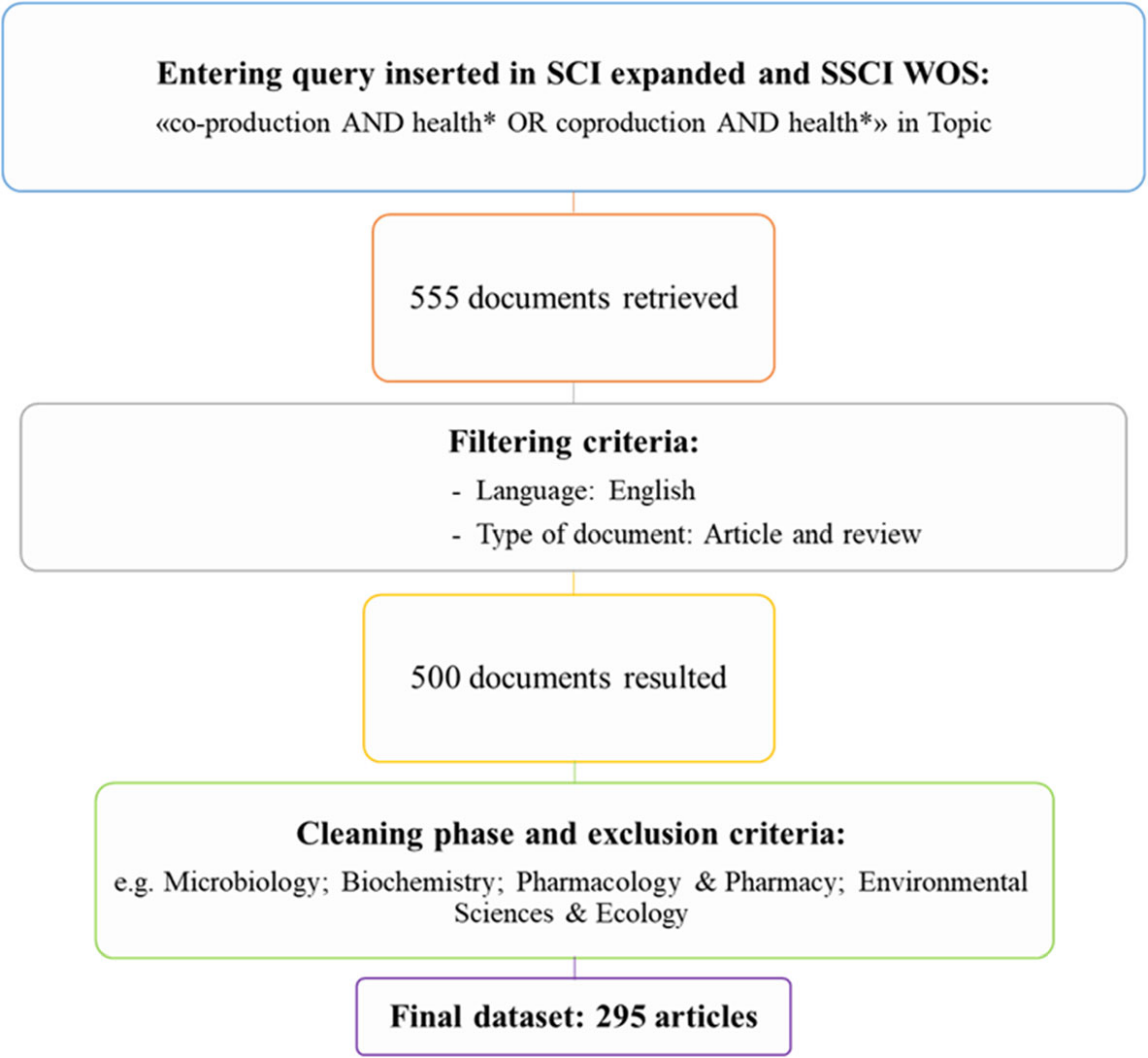
Data collection flow

The final sample is made up of 295 documents (see Additional file 1). Figure 1 summarizes the research design.

### Data analysis

Bibliometric techniques are based on the analysis of bibliographic attributes – also called “metadata” - of a document, such as authors, citations, collaboration, keywords, in order to have insights into a scientific field’s structure, social networks and relevant themes [35, 37]. In the present study, the bibliometric analysis was carried out using Bibliometrix, a free open source software application, supported by the R environment, which provides a set of tools for quantitative research in bibliometrics and scientometrics [37]. On the basis of the final sample retrieved by WoS in bibtex format, data were loaded and converted into R dataframe in Bibliometrix, in order to develop three main level of analysis: i) performance analysis; ii) collaboration analysis; and iii) science mapping.

#### Performance analysis

A performance analysis highlights the sample characteristics and measures its main performances by quantifying the research field (the number of published documents, the number of received citations), identifying the most important (most cited, most productive, etc) actors, and evaluating groups of scientific actors (countries, universities, departments, researchers) and the impact of their activity [38–41]. At this stage, a citation analysis was also performed. A citation analysis is based on the hypothesis that authors cite documents considered most important in the development of their research. Frequently cited studies are expected to have a greater influence on the research field than those less frequently cited [42, 43]. To ensure the accuracy of the data, the references were checked to ensure that they were written in the same way in all the documents.

#### Collaboration analysis

A scientific collaboration analysis was then carried out in order to highlight the most relevant relations between the actors (i.e. authors and countries) [44–47]. Scientific collaboration analysis is widely used in different strands of research [e.g. [48, 49]] in order to identify the social structure of the field. This is achieved via a social network analysis where the network’s nodes are the authors, their institution or country to which the institutions belong, and the edges (links) are established according to the nodes who co-authored an article.

#### Science mapping

The science mapping was performed through a co-citation analysis and co-word analysis. Science mapping “*is a spatial representation of how disciplines, fields, specialities, and individual papers or authors are related to one another*” [[40]:147].

The co-citation analysis was used to capture the intellectual structure of the field. Co-citation is defined as the frequency with which two documents are cited together in the literature and it assumes that documents are co-cited if they are conceptually close [50–52]. Given that it is a dynamic approach, it is often considered to be preferable to bibliographic coupling, which occurs when two documents have at least one reference in common. Its validity as a tool for exploring the intellectual structure of a scientific field has been stated in numerous studies [e.g. [50, 53]], and it is increasingly being used in all disciplines, including in the management field [e.g. [23, 36, 54]]. In the clustering derived from this analysis, the network nodes represent cited documents, whose size depends on the number of citations. The edges represent the co-citation relationship and their weights depend on the number of times that two documents have been cited jointly [50–52]. In this bibliometric analysis, a minimum degree of co-citation (equal to 2) has been set and a threshold of 50 network nodes has been considered. These settings ensure the clarity of the network, without compromising the validity of the results that did not have relevant deviations by increasing the size of the network.

A co-word analysis is based on the idea that the co-occurrence of key terms (i.e. abstract, title or keywords) describes the content of the documents [55]. This technique identifies and visualises clusters that represent semantic or conceptual groups of different topics treated in the research field. Using the approach developed by Cobo et al. [40, 41], the thematic clusters are visualised in a “strategic diagram” or map.

Moreover, the authors read the abstracts or full-text of papers when necessary to add relevant information to the results of the quantitative bibliometric analysis (e.g. in “Performance analysis” to add the methodological approach and main content of most cited papers and references; in “Co-citation analysis” to know the main contents of most important nodes and discuss the clusters). In the Additional file 2 the data (i.e. papers of the sample) and metadata (e.g. authors, citations, etc.) used in each analysis’ stage are detailed.

## Results

### Performance analysis: sample characteristics

The articles of the sample have been published from 1994 to 2019 (Table 1).

**Table 1:** Main sample information

Description	Results
Documents	295
Sources (Journals, Books, etc)	148
Period	1994–2019 (May)
Authors	1225
Author Appearances	1431
Single-authored documents	19
Authors per Document	4.15
Collaboration Index	4.37

**Fig. 2:**
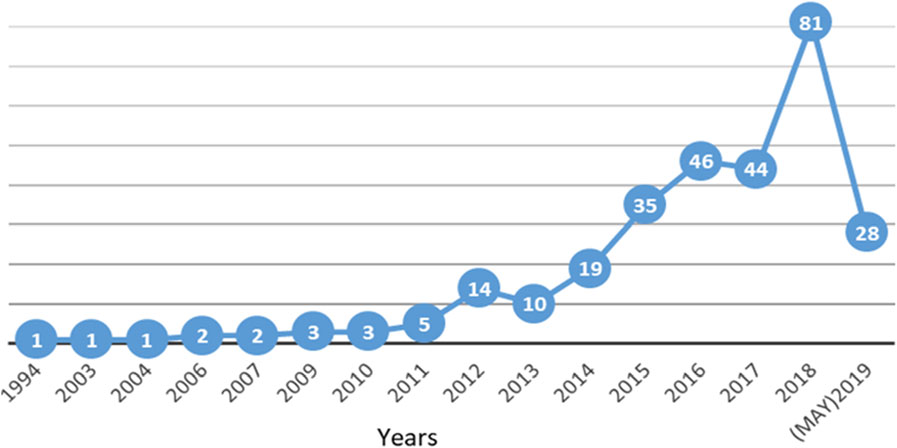
Annual scientific production

This research field is fairly recent with an annual scientific growth rate of nearly 25%. As shown in Fig. 2, the biggest increase has occurred in the last 4 years (about 80% of documents sourced); in 2018 the number of publications was more than double compared to 2015, and more than quadruple compared to 2014.

Authorship is very fragmented, with 1225 authors and a collaboration index (i.e. a Co-authors per Article index calculated only using the multi-authored article set) of 4.37. There is an average of 4.15 authors per document; only a few documents were written by one single author (19 articles, 6.4%). There is an average of 0.24 documents per author, and only 26 (2.1%) of authors have published more than three works, and 149 (12.2%) that have published more than two works. The most productive authors (Additional file 3) tend to be academics in medicine and nursing, with a lower incidence in psychology, social informatics and management.

The articles in the dataset were published in 148 journals, only 24 of which (16.2%) have published more than three articles and 46 (31%) more than two articles, with a mean of 1.9 article per journal. The most productive journals represent approximately 80% of the total number of documents retrieved; they belong to different areas of research – including medicine, management, business, social science - with nearly all in health fields according to WOS categories (Table 2). The top two journals cover the health field (i.e BMJ Open) and the public sector (i.e Public Management Review).

**Table 2:** Most productive journals

Sources	Articles	WOS Categories
BMJ Open	19	Medicine, General & Internal
Public Management Review	13	Management; Public Administration
Health Research Policy And Systems	12	Health Policy & Services
Health Expectations	9	Health Care Sciences & Services; Health Policy & Services; Public, Environmental & Occupational Health
Journal Of Health Organization And Management	9	Health Policy & Services
International Journal Of Mental Health Nursing	8	Nursing; Psychiatry
Implementation Science	7	Health Care Sciences & Services; Health Policy & Services
BMC Health Services Research	6	Health Care Sciences & Services
Social Policy & Administration	6	Development Studies; Public Administration; Social Issues; Social Work
BMC Public Health	5	Public, Environmental & Occupational Health
Health & Social Care In The Community	5	Public, Environmental & Occupational Health; Social Work
Journal Of Psychiatric And Mental Health Nursing	5	Nursing; Psychiatry
Journal Of Public Health	5	Public, Environmental & Occupational Health
Journal Of Service Research	5	Business
Social Science & Medicine	5	Public, Environmental & Occupational Health; Social Sciences, Biomedical

**Fig. 3:**
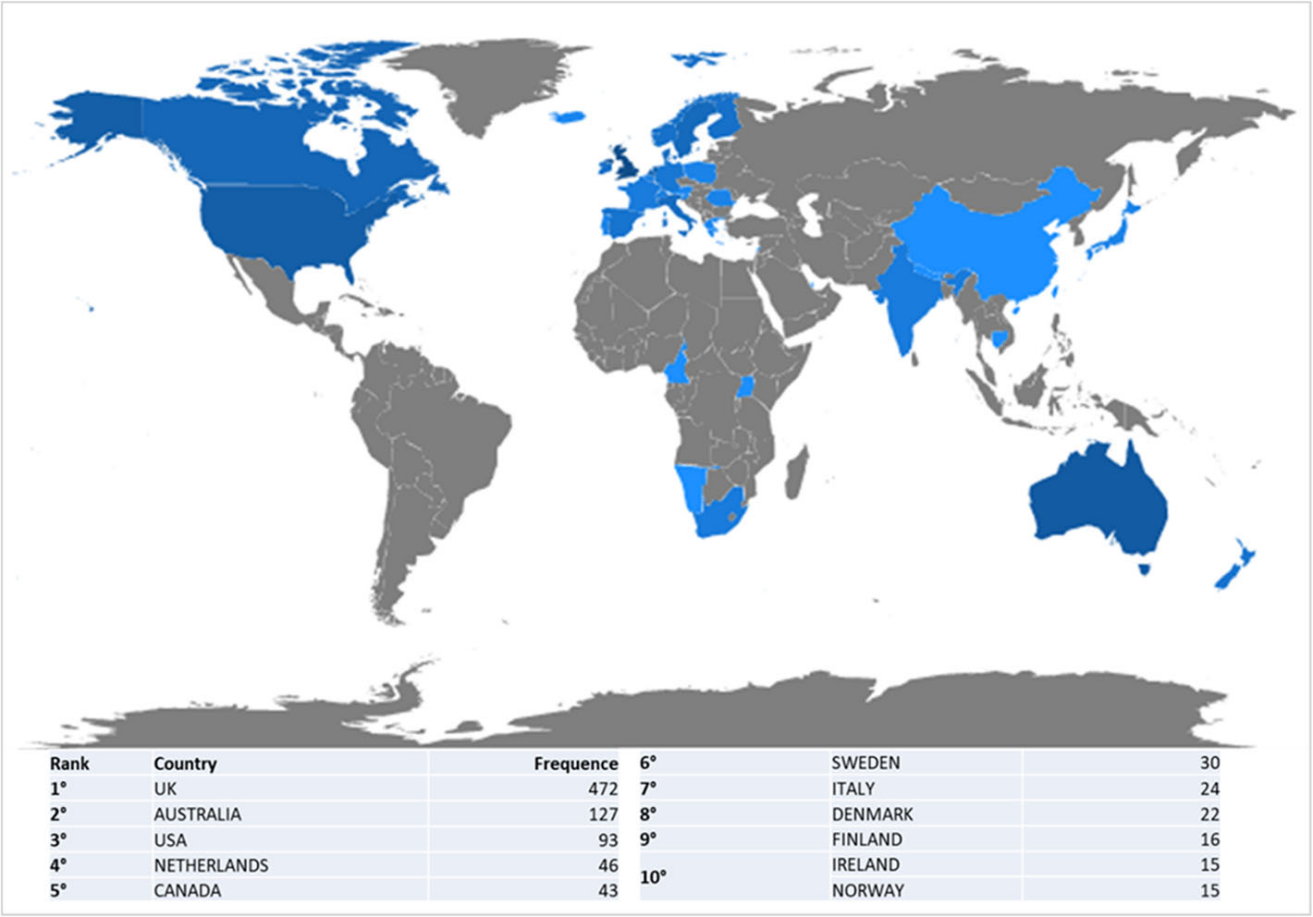
Scientific production by country. The map was generated through “Biblioshiny”, a shiny app providing a web-interface for Bibliometrix software (version 1.0, https://www.bibliometrix.org/Biblioshiny.html). Different shades of blue indicate different productivity rate: dark blue = high productivity; grey = no articles

The geographical distribution of papers – based on all authors’ affiliations - is concentrated in Anglo-Saxon countries (UK, USA, Australia, Canada and Ireland) and in other European countries (Netherlands, Sweden, Denmark, Italy, Finland and Norway) (Fig. 3).

The UK is the most productive country, where patient involvement in clinical practices has been a priority since the 1990s and new forms of organisation and delivery of healthcare services have been promoted by the NHS since the early 2000s [13, 56–58].

Table 3 shows the top 10 manuscripts per total citation, the first of which was published in 2006, when the scientific interest in the topic really began to take hold. The article with the highest number of total citations is the work by McColl-Kennedy et al. in 2012 [59], followed by the one written in 2015 by Voorberg et al. [5]) and by Carayon in 2006 [60]). The ranking changes slightly if annual citations are considered, i.e. the average number of citations received per year. These top 10 ranked articles consist of four conceptual papers, two reviews and four empirical papers. Interestingly, the empirical studies tend to try to develop theoretical or conceptual models/findings from the results: three adopt qualitative methods (i.e. the papers by McColl-Kennedy et al. [59]; Gillard et al. [61]; Mort et al. [62]) and one quantitative method (i.e. the work by Trummer et al. [63]). In these top ten papers, there is no homogeneity regarding the clinical field on which the study sample focused. Furthermore, one of the two reviews [5] is qualitative, i.e. a structured systematic review, the other uses a mixed method [64]. Four of the papers were published in health journals, followed by three in public administration and management and one in business, ergonomics and environmental sciences journals. It is not possible to identify reference theories for all ten papers, but four explicitly fit into the public management and administration literature, i.e. the works by Osborne et al. [6], by Batalden et al. [65], 2016; Vooberg et al. [5] and Dunston et al. [14]); the work of McColl-Kennedy et al. [59] refers to service management theories and the work of Gillard et al. [61] refers to Gittell’s theory on relational coordination. Two studies focus specifically on the co-production of knowledge [61, 64].

**Table 3:** Top manuscripts by citation

Paper_#Full reference	# list reference	Total Citation	Total citation per year	Type of article
McColl-Kennedy, J. R., Vargo, S. L., Dagger, T. S., Sweeney, J. C., & Kasteren, Y. V. (2012). Health care customer value cocreation practice styles. *Journal of Service Research*, 15(4), 370–389.	59	269	38,429	Empirical
Voorberg, W. H., Bekkers, V. J., & Tummers, L. G. (2015). A systematic review of co-creation and co-production: Embarking on the social innovation journey. *Public Management Review*, 17 (9), 1333–1357.	5	177	44,25	Review
Carayon, P. (2006). Human factors of complex sociotechnical systems. *Applied ergonomics*, 37 (4), 525–535.	60	144	11,077	Conceptual
Batalden, M., Batalden, P., Margolis, P., Seid, M., Armstrong, G., Opipari-Arrigan, L., & Hartung, H. (2016). Coproduction of healthcare service. *BMJ Quality & Safety*, 25, 509–517.	65	140	46,667	Conceptual
Fazey, I., Bunse, L., Msika, J., Pinke, M., Preedy, K., Evely, A. C., ... & Reed, M. S. (2014). Evaluating knowledge exchange in interdisciplinary and multi-stakeholder research. *Global Environmental Change*, 25, 204–220.	64	88	17,6	Review
Dunston, R., Lee, A., Boud, D., Brodie, P., & Chiarella, M. (2009). Co-production and health system reform–from re-imagining to re-making. *Australian Journal of Public Administration*, 68 (1), 39–52.	14	79	7,9	Conceptual
Trummer, U. F., Mueller, U. O., Nowak, P., Stidl, T., & Pelikan, J. M. (2006). Does physician–patient communication that aims at empowering patients improve clinical outcome?: A case study. *Patient education and counseling*, 61 (2), 299–306.	63	78	6	Empirical
Osborne, S. P., Radnor, Z., & Strokosch, K. (2016). Co-production and the co-creation of value in public services: a suitable case for treatment?. *Public Management Review*, 18 (5), 639–653.	6	74	24,667	Conceptual
Gillard, S., Simons, L., Turner, K., Lucock, M., & Edwards, C. (2012). Patient and public involvement in the coproduction of knowledge: reflection on the analysis of qualitative data in a mental health study. *Qualitative Health Research*, 22 (8), 1126–1137.	61	56	8	Empirical
Mort, M., Roberts, C., & Callén, B. (2013). Ageing with telecare: care or coercion in austerity?. *Sociology of health & illness*, 35 (6), 799–812.	62	38	3.45	Empirical

The top ten cited references (Table 4) are mainly co-production seminal works in the field of public services. Only two works, those by Dunston et al. [14] and by Batalden et al. [65] refer specifically to the health sector. The most cited works are the conceptual framework developed by Bovaird in 2007 [66], which is considered a milestone on co-production topic in public management, and the seminal work by Ostrom et al. in 1996 [9].

**Table 4:** Most cited references

Works #Full reference	#List reference	Citations	Type of publication	Approach (if applicable)
Bovaird, T. (2007). Beyond engagement and participation: User and community coproduction of public services. *Public administration review*, 67 (5), 846–860.	66	41	Academic paper	Conceptual
Ostrom, E. (1996). Crossing the great divide: coproduction, synergy, and development. *World development*, *24* (6), 1073–1087	9	31	Academic paper	Conceptual
*Batalden, M., Batalden, P., Margolis, P., Seid, M., Armstrong, G., Opipari-Arrigan, L., & Hartung, H. (2016). Coproduction of healthcare service. BMJ Quality & Safety, 25, 509–517.	65	28	Academic paper	Conceptual
*Dunston, R., Lee, A., Boud, D., Brodie, P., & Chiarella, M. (2009).Co-production and health system reform–from re-imagining to re-making. *Australian Journal of Public Administration*, *68* (1), 39–52	14	24	Academic paper	Conceptual
Braun, V., & Clarke, V. (2006). Using thematic analysis in psychology. *Qualitative research in psychology*, *3* (2), 77–101.	Not cited	23	Academic paper	Conceptual
Alford, J. (2009). *Engaging public sector clients: from service-delivery to co-production*. Springer.	4	21	Book	n.a.
Osborne, S. P., & Strokosch, K. (2013). It takes Two to Tango? Understanding the Co-production of Public Services by Integrating the Services Management and Public Administration Perspectives. *British Journal of Management*, 24, S31-S47.	70	19	Academic paper	Conceptual
Boyle, D., & Harris, M. (2009). The challenge of co-production. *London: New Economics Foundation*.	56	18	Discussion paper	Conceptual
Pestoff, V. (2006). Citizens and co-production of welfare services: Childcare in eight European countries. *Public management review*, 8 (4), 503–519.	22	16	Academic paper	Empirical
Alford, J. (2002). Why do public-sector clients coproduce? Toward a contingency theory. *Administration & Society*, *34* (1), 32–56.	71	15	Academic paper	Conceptual
Verschuere, B., Brandsen, T., & Pestoff, V. (2012). Co-production: The state of the art in research and the future agenda. *Voluntas: International Journal of Voluntary and Nonprofit Organizations*, 23 (4), 1083–1101.	7	15	Academic paper	Review

*Paper is also included in the sample considered for the current study

### Collaboration analysis

This analysis provides an overview of the scientific collaboration and research communities, with reference to different aggregation levels [47]. In this study, countries and authors were considered as units of analysis.

#### Cross-country collaboration

**Fig. 4:**
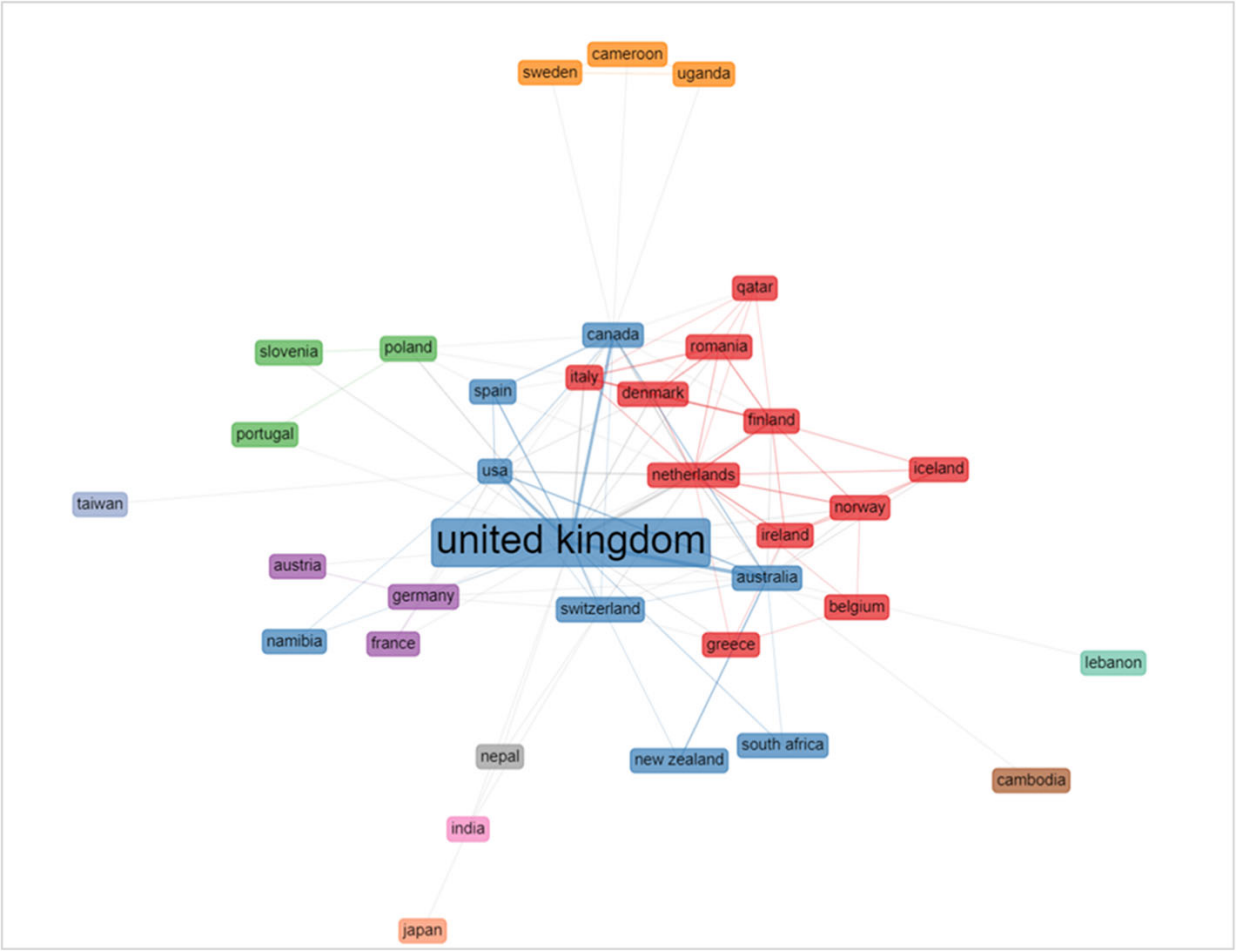
Country collaboration network. Density = 0.175; Degree Centralization = 0.54; Average path length = 2.074; Bibliometrix software attributes different colour to each cluster

The first analysis focused on cross-country collaboration (Fig. 4). The network has its central and most important node in the UK (betweenness centrality = 287.030); the other top nodes are the USA, Australia, Canada and the Netherlands.

The results are not totally surprising given the scientific production of each country highlighted in the performance analysis. However, further comments can be made regarding the multiple-country collaboration ratio of the top ten countries per publications i.e. the ratio between the number of multi-country collaborations and the total number of papers attributed based on the affiliation of the corresponding author (Table 5). The UK has the highest number of cross-country collaborations (20 multi-country collaborations), yet the UK’s multi-country publication (MCP) rate is only 13.9%, much lower than the ratios of Australia (40%), USA (25%) and Italy (22.2%).

**Table 5:** Production and collaborations of countries

Country	Articles	SCP	MCP	MCP_Ratio
United Kingdom	144	124	20	0.139
Australia	35	21	14	0.4
Netherlands	21	17	4	0.19
USA	20	15	5	0.25
Italy	9	7	2	0.222
Sweden	9	9	0	0
Denmark	7	6	1	0.143
Canada	6	5	1	0.167
Finland	6	5	1	0.167
Norway	5	4	1	0.2

*Legend: Country = country of the corresponding author’s affiliation; Articles = number of article per country of corresponding author’s affiliation; SCP = single country publication; MCP = multi country publication*

#### Collaboration among authors

**Fig. 5:**
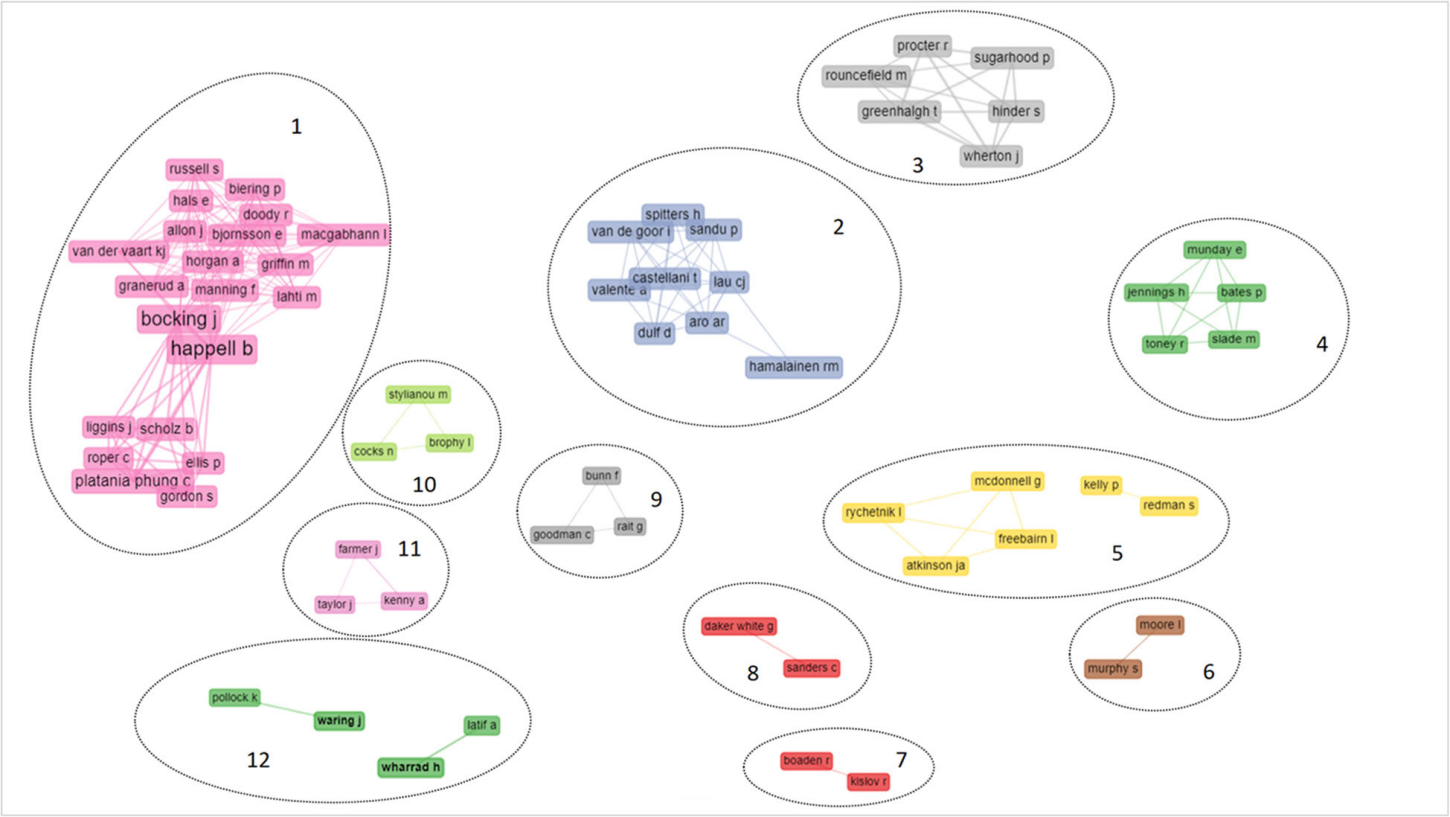
Co-authorship network. Density = 0.006; Degree centralization = 0.023; Average path length = 3.6; Bibliometrix software attributes different colour to each cluster

In order to understand any long-lasting collaborations among authors, the co-authorship network (Fig. 5) excluded one-shot collaborations (min.edge = 2). Only 66 out of 1225 authors, appeared to have collaborated with the same research group more than once, and they are clustered into twelve groups (see Additional file 4).

The clusters are characterised by differences in the disciplinary and professional background of the authors, geographical affiliation and research focus of the co-authors’ work.

Group 1 is the biggest and densest. It is made up of an academic cross-national network between Australian, New Zealand and northern European authors with a research focus on patient co-production in mental health.

Group 2 is a cross national authors’ network in Europe (i.e. Italy, Finland, Denmark, the Netherlands, Romania), focused on public health and policy.

Group 3 is composed of an interdisciplinary group of psychology, primary care and social informatics researchers, all with UK affiliation and focused on co-production in assisted-living technologies.

Group 4 is geographically limited to the UK, it includes both academics and practitioners and it is characterised by the research interest on patient co-production in mental health. .

Groups 5 and 6 share the same focus on public health and policy, but they have a different country’s affiliation. Group 5 is composed by Australian affiliation authors, group 6 by UK affiliation authors.

Groups 7 focuses on knowledge translation and is made up academic authors with UK affiliation. The same geographical characteristic can be found in Group 8, but the scientific focus is on health safety issues.

Group 9 is made up of primary care and nursing academics, with UK affiliation, specialised in dementia, comorbidity, integrated care and continuity of care for older people with complex health needs.

Group 10 refers to Australian academics and practitioners with an interest in research and evaluation in mental health.

Group 11 is composed by academics with an Australian university affiliation and a focus on service development and citizen participation in rural health.

Group 12 is made up of authors affiliated with universities in the UK in the field of co-production in medical education.

### The intellectual structure of the field: co-citation analysis

Intellectual structure is defined as “*the examined scientific domain’s research traditions, their disciplinary composition, influential research topics and the pattern of their interrelationships. These publications are the foundations upon which current research is being carried out and contain fundamental theories, breakthrough early works and methodological canons of the field*” [[35]:438]. Intellectual structure is investigated through a co-citation analysis which verifies the presence and frequency of the co-citations in a dataset and identifies which clusters of citations are conceptually related and how relevant they are [50, 53].

**Fig. 6:**
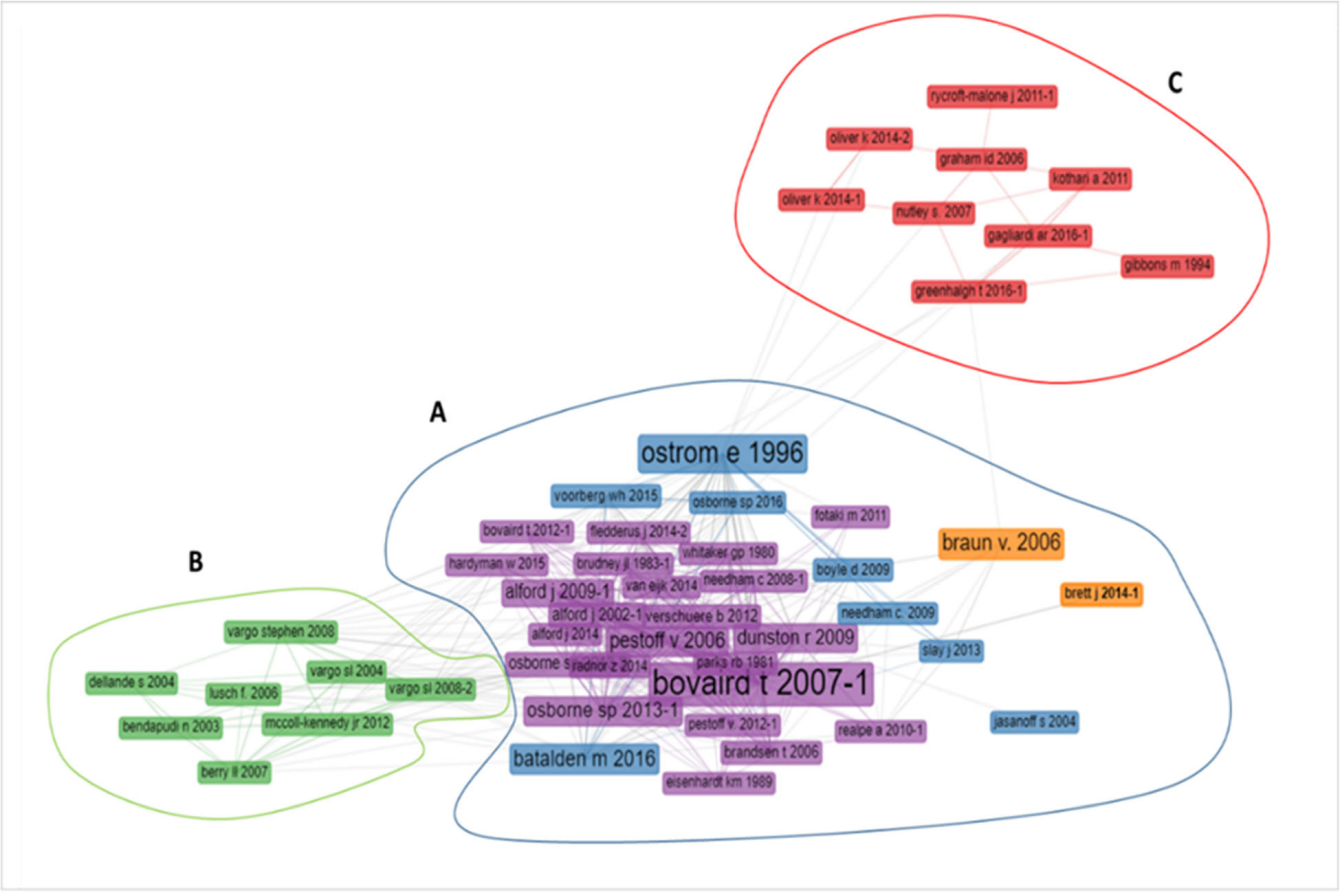
Co-citation network of references. Density = 0.006; Degree centralization = 0.157; Average path length = 3.116; Bibliometrix software attributes different colour to each cluster

The co-citation analysis (Fig. 6 and Additional file 5) was carried out with a minimum degree of co-citation equal to 2 and a threshold of 50 network nodes. This was designed on the basis of the number of papers and the fragmentation of the field as well as to ensure the readability of the network. Other options were also tested, without significantly different results.

The findings show a network whose most central and important nodes are represented by “Bovaird t 2007-1”^1^ (betweenness centrality = 103.337) [66] and “Ostrom e 1996” (betweenness centrality = 170.150) [9], that are also the two most cited documents in the database (see also the above Table 4). The study by Bovaird [66] was one of the first to report a conceptual framework of co-production. Co-production is described as a “*revolutionary concept in public service*” [[66]:846] and the framework maps the various interactions that can be established among public providers, service users and their communities. It identifies the various stages where co-production can take place: planning, design, commissioning, management, delivery, monitoring, and evaluation. Bovaird also provides some case studies that give insights into the benefits and challenges of co-production.

Using an institutional-economic approach, Ostrom [9] analyses co-production as a way to overcome the divide identified between “*a market-based logic of development and the traditional theories of public administration*” [[9]:1073]. Her two case studies suggest that co-production into polycentric and synergic systems between government agencies and citizens is crucial in order to reach higher levels of welfare in developing countries.

The co-citation analysis revealed three main clusters.

*A: “Public administration and management”.* This is the largest group (33 works) with the densest ties and the most top nodes in the entire network. Seminal and early contributions are represented by “Parks RB 1981” [67], “Whitaker GP 1980” [68] and “Ostrom e 1996” [9]. The cluster includes some political science and economics studies [e.g. [9, 67]] and a predominance of public administration and management works [e.g. [66, 69]]. These studies consider co-production as a tool of public policy aimed at improving the efficiency and effectiveness of public services. “*Coproduction is one way that synergy between what a government does and what citizens do can occur*.” [[9]:1079]. The prevailing approach is top-down, strongly focused on the public service provider. Co-production is seen as something “*to be consciously built into public services*” [[70]:S35] and thus the discussion is centred on “*the ways in which service user participation can be ‘added into’ the process of service planning and production*” [[70]:S34]. Three types of possible co-producers are identified in the public service regime (clients, citizens and volunteers) and differences with co-production in the private sector are discussed [4, 71].

The papers in this cluster detail the conceptualization of co-production in the public sector, analyse its pros and cons, as well as how its implementation can be incentivised and managed. Seven of these specifically investigate the health sector, seen as a key area for the implementation of co-production practices [e.g. [14, 65, 72]]. Co-production is seen as a promising tool for dealing with challenges in the sector such as increasing and changing demands with a simultaneous decrease in resources [14]. The number of elderly people, with multi-faceted needs and high expectations, as well as the rates of chronic diseases are growing [1, 73]. This puts the onus on healthcare systems to contain costs without detracting from the high quality of care. Rising hospitalization costs are forcing healthcare administrators to reduce the length of hospital stays and the readmission rate, making it necessary to build relational models in which the patient feels part of the healthcare team and willing and able to continue self-care after discharge. Co-production can help to ensure both the improvement in the service and sustainability in the system [14], taking into account possible limitations and challenges [72].

*B: “Service management”.* This consists of eight documents from the service management and marketing literature. In this cluster there is little crossover with other disciplines. In terms of service management, these works focus on the (co-)creation of value for/with the customers/users. The internal top nodes are represented by “Vargo SL 2008-2” [74], “Vargo Stephen 2008” [75], and “Bendapudi N 2003” [76]. From this perspective, organisations consider customers as an important resource, quasi-employees, who carry out part of the service delivery. This concept of customer participation then leads to a service-dominant (S-D) approach where service is the common denominator in exchange, and within its delivery, the customer is a co-creator of value both for the firm and her/himself [75, 77, 78]. From this perspective, some studies refer to a specific topic, such as customer behaviours, practices and psychological implications [e.g. 76, 59]. Given these theoretical assumptions, scholars point out that service management and a marketing perspective have much to offer the analysis and interpretation of a “*critically important and intellectually challenging*” [[79]:111] sector, i.e. healthcare. Healthcare is considered a fertile field for empirical research, given that “*no other service sector affects the quality of life more than health care. No other service commands more resources or is more challenged as it faces the future”* [[79]:121]. In line with the focus of this cluster on service management rather than on the public domain, the studies refer to private healthcare organisations [59, 79, 80].

The visual representation of the co-citation network shows the presence of numerous ties and bridges-nodes (connecting nodes between two clusters), that are “Osborne SP 2013-1” [70], “Osborne SP 2013-2” [81]; “Hardyman W 2015” [82] between the two clusters. In fact, there has recently been a crossover between public management and service management in studies that discuss how the public management literature can benefit from the service paradigm, for example in the development of a public service-dominant approach [70, 81, 83] or the work on the co-creation of public value [84].

*C: “co-production of knowledge*”. This cluster has fewer internal and external ties. The cluster focuses on the co-production of knowledge in its specific meaning of knowledge translation between researchers and decision-makers (clinicians, managers, policy-makers, etc.) “*for the purpose of engaging in a mutually beneficial research project or program of research to support decision-making*” [[85]:11]. The user’s or patient’s perspective is, therefore, almost neglected, with the partial exception of “Greenhalgh T 2016-1” [86], who focus on community-academic partnerships. The top nodes are represented by “Gagliardi AR 2016-1” [85] and “Graham ID 2006” [87], which are both literature reviews as well as most of the other works in cluster. Moreover, unlike the other clusters, six out of the nine studies are specifically on the health sector.

### The thematic map: co-word analysis

A co-word analysis of author keywords (min. frequency = 3; number of words = 300) helps to define a map of the main themes of the field. In order to avoid deviant results, the dataset was screened before being submitted to analysis by the software. Generic keywords inserted in the search query were eliminated (i.e. *coproduction; co-production; health*); ii) the spelling of keywords was harmonized (e.g., plural or singular; American or English style; with hyphen or without hyphen).

**Fig. 7:**
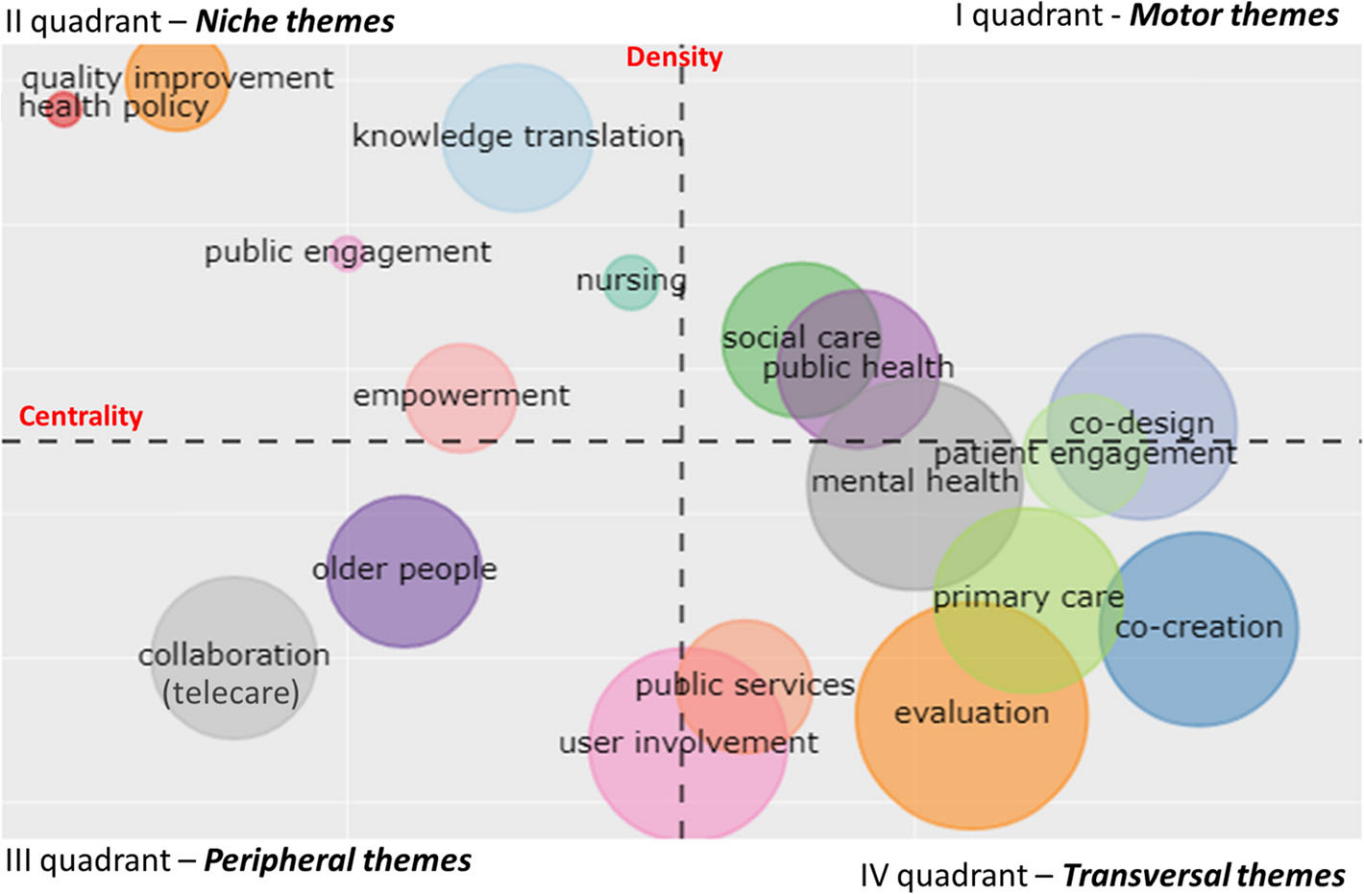
Thematic map of the field

Using the visualization technique proposed by Cobo et al. [40, 41], a strategic thematic map was developed, plotting the themes into four quadrants (clusters of keywords) according to their centrality and density rank values along two axes. The centrality measures the degree of interaction of a network with other networks and is considered “*as a measure of the importance of a theme in the development of the entire research field analysed*.” [[40]:150]. The density measures the internal strength of the network and identifies the degree of development of a theme. The size of the cluster is given by the number of occurrences of the keywords that it contains and therefore by the number of linked papers. The label chosen by the software corresponds to the predominant keyword (Fig. 7).

For each quadrant, clusters with a higher number of related papers are discussed.

- *Motor themes* (first quadrant): these are well developed themes that are key to the structure of the research field, and are characterized by high centrality and high density. There were few “motor” themes - “public health”, “social care” and “co-design” - and they are at the axis of the fourth quadrant. Social care mainly refers to the co-production and personalisation of community health and the delivery of well-being services [88]. The focus is above all on the weakest segments of the community or on integration difficulties [89, 90]. Confirming the evidence on collaboration among authors, “public health” is one of the most explored fields, both in terms of the co-production of knowledge [91, 92] and service design and/or delivery [93, 94]. “Co-design” is a specific phase of co-production referring to activities that involve “*the experience of users and their communities” into the creation, planning, or arrangements of public services* [[12]:772]. Examples of co-design in our dataset were related to the improvement of hospital services [17], public health [95], integrated care [96], and assistive technology [97].
- *Niche themes* (second quadrant): these are well developed and very specialised themes, but marginal in the overall field. “Knowledge translation” appears to be a most recurrent theme in this quadrant. Its internal specialization and external marginality were evident also in the intellectual structure (i.e. cluster “co-production of knowledge”), where it was scarcely connected with other clusters. Knowledge translation concerns the partnership between researchers and practitioners-decision makers aimed at reducing the gap between research and practice [98, 99]. The concept of “empowerment” is strictly related to co-production [20], and relates to patient self-confidence and the understanding of their role. Some authors considered empowerment as a determinant of co-production, given that co-production requires “*that a degree of confidence exists for any patient to feel sufficiently empowered to actively engage*.” [[58]:551]. Others argue that it is engagement and co-production that boost empowerment because they increase confidence and give users a sense of influence or control [100]. The theme “quality improvement” focuses on the improvement of health services, which is achieved primarily through co-production and personalisation of patient care [101, 102]. The “Nursing” cluster highlights the recognition of the importance of the role of nurses in health service delivery, thanks to their closeness to end-users and the role played by patients (experts by experience) in improving nursing education and training [103].
- *Peripheral themes* (third quadrant): this comprises both emerging and declining themes, characterised by low density (under developed) and centrality (marginal). This quadrant includes care for the elderly, who are a critical and weak group in the population in terms of chronicity and comorbidity and therefore with greater health and social care needs [104]. In this context, there are frequent examples of co-production through technology (e.g. telecare or telehealth), which facilitate the monitoring of the patient’s health conditions, the possibility of providing services at home, and the involvement of patients and caregivers in the process of care [105].
- *Transversal and general, basic themes* (fourth quadrant): these are themes with a high centrality and low density, which are important for the co-production field, but are still not well developed. They mostly concern umbrella themes, such as “user involvement”, “patient engagement”, which the most recent psychological and organizational literature prefers to the term “empowerment”. The term “co-creation” is now very widespread, but not very focused. This quadrant includes papers, whose intellectual structure mainly refers to the managerial field, both public and service, with a strong focus on the patient as the key-actor in the process. Two main themes refer to the most relevant health disciplines where co-production has been widely applied. The first is “mental health”, both with reference to co-production of knowledge [61] and services [106]. Several articles investigate the recovery college model, which as an alternative to the traditional clinical model aims to treat and reduce symptoms; it is inspired by the broader principles of mental well-being, recovery and co-production [107]. Indeed, in these colleges, the training courses, whose participants are not only patients, but also caregivers and staff, are co-designed and co-delivered by medical personnel and experts by experience, whose contribution is recognized as having a strong impact on the effectiveness of the model [107, 108]. The second area is “Primary care” which covers studies referring to the management of long-term conditions, where co-production and self-management become necessary both for health providers and patients and their caregivers [109, 110]. Moreover, the presence of “evaluation” as a theme highlights that it is considered central for the overall understanding of the co-production practices. Several papers have reported the potential of the co-delivery of healthcare services, e.g., peer support groups, nurse- family partnerships [106, 111–113]. Nevertheless, evidence of a clear link to correlate co-production to greater benefits for those involved is, at best, weak [7]. When explored, the impact is measured through subjective methods, such as satisfaction questionnaires or interviews with patients, staff or caregivers [108, 114].

## Discussion

This bibliometric analysis provides a comprehensive picture of the structure and the development of co-production in the healthcare sector.

Although there have been some valuable qualitative reviews [16], to the best of our knowledge this is the first study to use a quantitative approach and cover the most recent published literature. The analysis shows that academic interest in co-production has increased considerably with an annual growth rate of nearly 25%: publications increased four-fold between 2014 and 2018 (almost 300 articles were included in the current analysis, compared to 65 in a previous review).

Despite this fast-growing interest in co-production in healthcare, the field is still very fragmented. The 295 articles retrieved are split between 1225 authors and 148 sources, of which only 12.2% of authors and 31% of journals have published more than two works. The top authors mainly belong to the area of medicine and nursing, with a lower incidence of other specialisations (psychology, social informatics and management).

This fragmentation is also highlighted by the collaboration analysis, which shows very few long-lasting collaborations. Authors collaborate frequently (with an average of 4.15 authors per document), but generally only once.

The field appears to be concentrated in the EU and Anglo-Saxon countries, with an absolutely central role of the United Kingdom, as shown by the performance analysis as well as the collaboration analysis. Although the UK has the highest productivity rate, the collaborations are mostly with other authors located in the UK. The proliferation of co-production research in these countries could be justified by the fact that they were early adopters of patient involvement clinical practices and there is also a strong commitment to co-production by the government and the NHS, in order to cut costs and improve the efficiency of public services [1, 13, 56, 57].

The intellectual structure of health co-production consists of two main subfields (clusters), i.e. public management and service management perspectives. Public services have rarely featured in service management research, which is surprising given the discipline’s long intellectual history in the concepts of co- production, value-in-exchange and value-in-use. The co-citation analysis revealed a recent but growing crossflow between the two disciplines. In particular, public management academics have investigated how a service-dominant approach [70, 82] and value co-creation [84] can effectively be applied to explain the dynamics of co-production in public sector.

The thematic map developed through the co-word analysis helps to identify the most consolidated themes and to provide evidence on the emerging ones. The analysis found few well-developed and central terms and these tended to refer to traditional areas of co-production (e.g. public health, social care). This confirms the relative low scientific maturity of co-production applied to health services. On the other hand, the analysis revealed some emerging themes related to social and health phenomena (e.g. the elderly and chronic diseases), the use of technologies, and the recent patient-centred approach to care (patient involvement / engagement).

## Conclusion

The bibliometric method adopted in this study was very useful for investigating and providing a comprehensive picture of co-production in the health field, especially due to the various techniques used (performance analysis, collaboration analysis and science mapping). Nonetheless, the paper has some limitations, mostly concerning the methodological issues adopted. Only using “co-production” as keyword in the search clearly produced a smaller dataset than might otherwise have been possible; yet it meant that this concept could be analysed in its narrow meaning in combination with related and similar concepts. Although a bibliometric method is objective and reproducible, it also implies a less detailed understanding than qualitative techniques. For example, the citation and co-citation analyses show the most cited references, but do not reveal the reason for the citation. Again, the conceptual map highlights the main themes, but does not allow for an in-depth analysis of the contents of each paper.

The results help to identify avenues for future research. While the branch of knowledge co-production and service co-design (hence the user’s participation in the research phase) seems to be more developed in terms of empirical evidence, the research on co-delivery and co-management still seems to be in an embryonic and more theoretical stage. Specifically, little has been produced on how the organisation of health services should change or adapt in order to consider the patient as a partner in designing, monitoring, delivering and evaluating a work practice. Even less investigated is the theme of the impact of co-production on providers and on patients. The few theoretical studies and even fewer empirical studies on this topic adopt predominantly a mono-dimensional and a mono-stakeholder approach. Specifically, psychological-social impacts are assessed, above all in terms of patient satisfaction.

Besides, even the medical research on home therapy practices – which represent a form of “co-production” - tends to focus only on the economic and clinical impacts [115–117]. At present, the presence and the description of co-produced practices is often accepted a priori as a successful output. The implementation of the activity is, therefore, confused with its result or, even if identified, it is based on self-reported data, widely used in management studies, but often applied or reported with little methodological rigor.

What does embracing co-production really mean? What value does it create? In what phase can co-production create the most value? How can this value be measured? Future research could try to answer these questions by trying to provide a clearer and unambiguous understanding of the construct (which initiatives should be interpreted as authentic co-production processes?) and what its results are.

## Supplementary information

**Additional file 1.** List of dataset papers

**Additional file 2.** Description of metadata per performed analysis

**Additional file 3.** Most productive authors

**Additional file 4.** List of authors’ collaboration

**Additional file 5.** List of intellectual structure clusters

## Data Availability

All data generated or analysed during this study are included in this published article (and its supplementary information files).

## Abbreviations

WoS: Web of science
SCI expanded: Science citation index expanded
SSCI: Social science citation index
EU: European union
UK: United Kingdom
USA: United States of America
NHS: National health service (UK)
MCP: Multi-country publication
SCP: Single country publication

## Footnotes

1 In this paragraph, the label of nodes contained in the network (Fig. 6) is followed by the citation in square brackets.
