# Supplementary material for: Co-production in health policy and management: a comprehensive bibliometric review
Source: BMC Health Serv Res. 2020 Jun 5;20:504. doi: 10.1186/s12913-020-05241-2 (PMC7275357; doi:10.1186/s12913-020-05241-2)
Supplement: Supplementary file 1 — Additional file 1. List of dataset papers [file 12913_2020_5241_MOESM1_ESM.docx]

***Additional file 1: List of papers in dataset***

| **Author(s)** | **Title** | **Source** | **Year** |
| --- | --- | --- | --- |
| Abma Ta;Voskes Y;Widdershoven G | Participatory Bioethics Research And Its Social Impact: The Case Of Coercion Reduction In Psychiatry | *Bioethics* | 2017 |
| Abma Ta;Baur Ve | User Involvement In Long-Term Care. Towards A Relational Care-Ethics Approach | *Health Expectations* | 2015 |
| Adams Sa | Maintaining The Collision Of Accounts: Crowdsourcing Sites In Health Care As Brokers In The Co-Production Of Pharmaceutical Knowledge | *Information Communication \& Society* | 2014 |
| Addis S;Holland Hart D;Edwards A;Neal Rd;Wood F | Implementing Prudent Healthcare In The Nhs In Wales, What Are The Barriers And Enablers For Clinicians? | *Journal Of Evaluation In Clinical Practice* | 2019 |
| Ahuja As;Williams R | Telling Stories: Learning From Patients' And Families' Experiences Of Specialist Child And Adolescent Mental Health Services | *International Journal Of Consumer Studies* | 2010 |
| Alford J;Yates S | Co-Production Of Public Services In Australia: The Roles Of Government Organisations And Co-Producers | *Australian Journal Of Public Administration* | 2016 |
| Allen K;Needham C;Hall K;Tanner D | Participatory Research Meets Validated Outcome Measures: Tensions In The Co-Production Of Social Care Evaluation | *Social Policy \& Administration* | 2019 |
| Andreassen Ta | From Democratic Consultation To User-Employment: Shifting Institutional Embedding Of Citizen Involvement In Health And Social Care | *Journal Of Social Policy* | 2018 |
| Andritsos Da;Tang Cs | Incentive Programs For Reducing Readmissions When Patient Care Is Co-Produced | *Production And Operations Management* | 2018 |
| Anstiss T | Motivational Interviewing In Primary Care | *Journal Of Clinical Psychology In Medical Settings* | 2009 |
| Askheim Op;Christensen K;Fluge S;Guldvik I | User Participation In The Norwegian Welfare Context: An Analysis Of Policy Discourses | *Journal Of Social Policy* | 2017 |
| Baim Lance A;Tietz D;Lever H;Swart M;Agins B | Everyday And Unavoidable Coproduction: Exploring Patient Participation In The Delivery Of Healthcare Services | *Sociology Of Health \& Illness* | 2019 |
| Baim Lance A;Tietz D;Schlefer M;Agins B | Health Care User Perspectives On Constructing, Contextualizing, And Co-Producing ``Quality Of Care'' | *Qualitative Health Research* | 2016 |
| Baines R;Donovan J;De Bere Sr;Archer J;Jones R | Responding Effectively To Adult Mental Health Patient Feedback In An Online Environment: A Coproduced Framework | *Health Expectations* | 2018 |
| Barbieri N;Gallego R;Morales E;Rodriguez Sanz M;Palencia L;Isabel Pasarin M | Measuring And Analysing Community Action For Health: An Indicator-Based Typology And Its Application To The Case Of Barcelona | *Social Indicators Research* | 2018 |
| Batalden M;Batalden P;Margolis P;Seid M;Armstrong G;Opipari Arrigan L;Hartung H | Coproduction Of Healthcare Service | *Bmj Quality \& Safety* | 2016 |
| Beckett K;Farr M;Kothari A;Wye L;Le May A | Embracing Complexity And Uncertainty To Create Impact: Exploring The Processes And Transformative Potential Of Co-Produced Research Through Development Of A Social Impact Model | *Health Research Policy And Systems* | 2018 |
| Bekker M;Van Egmond S;Wehrens R;Putters K;Bal R | Linking Research And Policy In Dutch Healthcare: Infrastructure, Innovations And Impacts | *Evidence \& Policy* | 2010 |
| Bekker Mpm;Mays N;Helderman Jk;Petticrew M;Jansen Mwj;Knai C;Ruwaard D | Comparative Institutional Analysis For Public Health: Governing Voluntary Collaborative Agreements For Public Health In England And The Netherlands | *European Journal Of Public Health* | 2018 |
| Berends L;Ritter A;Chalmers J | Collaborative Governance In The Reform Of Western Australia's Alcohol And Other Drug Sector | *Australian Journal Of Public Administration* | 2016 |
| Bolton M;Moore I;Ferreira A;Day C;Bolton D | Community Organizing And Community Health: Piloting An Innovative Approach To Community Engagement Applied To An Early Intervention Project In South London | *Journal Of Public Health* | 2016 |
| Bosco A;Schneider J;Coleston Shields Dm;Orrell M | Dementia Care Model: Promoting Personhood Through Co-Production | *Archives Of Gerontology And Geriatrics* | 2019 |
| Boswell J;Settle C;Dugdale A | Who Speaks, And In What Voice? The Challenge Of Engaging `Thepublic' In Health Policy Decision-Making | *Public Management Review* | 2015 |
| Bowen Kj;Miller Fp;Dany V;Graham S | The Relevance Of A Coproductive Capacity Framework To Climate Change Adaptation: Investigating The Health And Water Sectors In Cambodia | *Ecology And Society* | 2015 |
| Boydell Km;Dew A;Hodgins M;Bundy A;Gallego G;Iljadica A;Lincoln M;Pignatiello A;Teshima J;Willis D | Deliberative Dialogues Between Policy Makers And Researchers In Canada And Australia | *Journal Of Disability Policy Studies* | 2017 |
| Braybrook De;Witty Kr;Robertson S | Men And Lung Cancer: A Review Of The Barriers And Facilitators To Male Engagement In Symptom Reporting And Screening | *Journal Of Mens Health* | 2011 |
| Brewster L;Aveling El;Martin G;Tarrant C;Dixon Woods M;Collaboration Writing Comm Cwc | What To Expect When You're Evaluating Healthcare Improvement: A Concordat Approach To Managing Collaboration And Uncomfortable Realities | *Bmj Quality \& Safety* | 2015 |
| Brocklehurst Pr;Mckenna G;Schimmel M;Kossioni A;Jerkovic Cosic K;Hayes M;Da Mata C;Muller F | How Do We Incorporate Patient Views Into The Design Of Healthcare Services For Older People: A Discussion Paper | *Bmc Oral Health* | 2018 |
| Brophy L;Bruxner A;Wilson E;Cocks N;Stylianou M | How Social Work Can Contribute In The Shift To Personalised, Recovery-Oriented Psycho-Social Disability Support Services | *British Journal Of Social Work* | 2015 |
| Brown M;Hoyle L;Karatzias T | The Experiences Of Family Carers In The Delivery Of Invasive Clinical Interventions For Young People With Complex Intellectual Disabilities: Policy Disconnect Or Policy Opportunity? | *Journal Of Clinical Nursing* | 2016 |
| Bucci S;Schwannauer M;Berry N | The Digital Revolution And Its Impact On Mental Health Care | *Psychology And Psychotherapy-Theory Research And Practice* | 2019 |
| Buckley Bjr;Thijssen Dhj;Murphy Rc;Graves Lef;Whyte G;Gillison Fb;Crone D;Wilson Pm;Watson Pm | Making A Move In Exercise Referral: Co-Development Of A Physical Activity Referral Scheme | *Journal Of Public Health* | 2018 |
| Buettgen M;Schumann Jh;Ates Z | Service Locus Of Control And Customer Coproduction: The Role Of Prior Service Experience And Organizational Socialization | *Journal Of Service Research* | 2012 |
| Bunn F;Goodman C;Manthorpe J;Durand Ma;Hodkinson I;Rait G;Millac P;Davies Sl;Russell B;Wilson P | Supporting Shared Decision-Making For Older People With Multiple Health And Social Care Needs: A Protocol For A Realist Synthesis To Inform Integrated Care Models | *Bmj Open* | 2017 |
| Bunn F;Burn Am;Robinson L;Poole M;Rait G;Brayne C;Schoeman J;Norton S;Goodman C | Healthcare Organisation And Delivery For People With Dementia And Comorbidity: A Qualitative Study Exploring The Views Of Patients, Carers And Professionals | *Bmj Open* | 2017 |
| Burton Cr;Malone Jr;Robert G;Willson A;Hopkins A | Investigating The Organisational Impacts Of Quality Improvement: A Protocol For A Realist Evaluation Of Improvement Approaches Drawing On The Resource Based View Of The Firm | *Bmj Open* | 2014 |
| Cairney P;Oliver K | Evidence-Based Policymaking Is Not Like Evidence-Based Medicine, So How Far Should You Go To Bridge The Divide Between Evidence And Policy? | *Health Research Policy And Systems* | 2017 |
| Carayon P | Human Factors Of Complex Sociotechnical Systems | *Applied Ergonomics* | 2006 |
| Cepiku D;Giordano F | Co-Production In Developing Countries: Insights From The Community Health Workers Experience | *Public Management Review* | 2014 |
| Chadborn N;Craig C;Sands G;Schneider J;Gladman J | Improving Community Support For Older People's Needs Through Commissioning Third Sector Services: A Qualitative Study | *Journal Of Health Services Research \& Policy* | 2019 |
| Chambers M;Mcandrew S;Nolan F;Thomas B;Watts P;Kantaris X | Service User Involvement In The Coproduction Of A Mental Health Nursing Metric: The Therapeutic Engagement Questionnaire | *Health Expectations* | 2017 |
| Cheetham M;Wiseman A;Khazaeli B;Gibson E;Gray P;Van Der Graaf P;Rushmer R | Embedded Research: A Promising Way To Create Evidence-Informed Impact In Public Health? | *Journal Of Public Health* | 2018 |
| Christensen K;Pilling D | User Participation Policies In Norway And England - The Case Of Older People And Social Care | *Journal Of Social Policy* | 2019 |
| Clarke D;Jones F;Harris R;Robert G;Collaborative Rehabil Environm Cre | What Outcomes Are Associated With Developing And Implementing Co-Produced Interventions In Acute Healthcare Settings? A Rapid Evidence Synthesis | *Bmj Open* | 2017 |
| Clarke J;Waring J;Timmons S | The Challenge Of Inclusive Coproduction: The Importance Of Situated Rituals And Emotional Inclusivity In The Coproduction Of Health Research Projects | *Social Policy \& Administration* | 2019 |
| Clifton A;Repper J;Banks D;Remnant J | Co-Producing Social Inclusion: The Structure/Agency Conundrum | *Journal Of Psychiatric And Mental Health Nursing* | 2013 |
| Corburn J;Curl S;Arredondo G;Malagon J | Health In All Urban Policy: City Services Through The Prism Of Health | *Journal Of Urban Health-Bulletin Of The New York Academy Of Medicine* | 2014 |
| Cramm Jm;Nieboer Ap | The Changing Nature Of Chronic Care And Coproduction Of Care Between Primary Care Professionals And Patients With Copd And Their Informal Caregivers | *International Journal Of Chronic Obstructive Pulmonary Disease* | 2016 |
| Cramm Jm;Nieboer Ap | A Longitudinal Study To Identify The Influence Of Quality Of Chronic Care Delivery On Productive Interactions Between Patients And (Teams Of) Healthcare Professionals Within Disease Management Programmes | *Bmj Open* | 2014 |
| Curnutte M;Testa G | Consuming Genomes: Scientific And Social Innovation In Direct-To-Consumer Genetic Testing | *New Genetics And Society* | 2012 |
| Dalgarno M;Oates J | The Meaning Of Co-Production For Clinicians: An Exploratory Case Study Of Practitioner Trainers In One Recovery College | *Journal Of Psychiatric And Mental Health Nursing* | 2018 |
| Davies J;Sampson M;Beesley F;Smith D;Baldwin V | An Evaluation Of Knowledge And Understanding Framework Personality Disorder Awareness Training: Can A Co-Production Model Be Effective In A Local Nhs Mental Health Trust? | *Personality And Mental Health* | 2014 |
| Daykin N;Gray K;Mccree M;Willis J | Creative And Credible Evaluation For Arts, Health And Well-Being: Opportunities And Challenges Of Co-Production | *Arts \& Health* | 2017 |
| De Andrade M;Angus K;Hastings G | Teenage Perceptions Of Electronic Cigarettes In Scottish Tobacco-Education School Interventions: Co-Production And Innovative Engagement Through A Pop-Up Radio Project | *Perspectives In Public Health* | 2016 |
| De Witte K;Geys B | Citizen Coproduction And Efficient Public Good Provision: Theory And Evidence From Local Public Libraries | *European Journal Of Operational Research* | 2013 |
| Del Bene D;Scheidel A;Temper L | More Dams, More Violence? A Global Analysis On Resistances And Repression Around Conflictive Dams Through Co-Produced Knowledge | *Sustainability Science* | 2018 |
| Dent M;Pahor M | Patient Involvement In Europe - A Comparative Framework | *Journal Of Health Organization And Management* | 2015 |
| Derges J;Kidger J;Fox F;Campbell R;Kaner E;Taylor G;Mcmahon C;Reeves L;Hickman M | `Drinkthink' Alcohol Screening And Brief Intervention For Young People: A Qualitative Evaluation Of Training And Implementation | *Journal Of Public Health* | 2018 |
| Derges J;Clow A;Lynch R;Jain S;Phillips G;Petticrew M;Renton A;Draper A | `Well London' And The Benefits Of Participation: Results Of A Qualitative Study Nested In A Cluster Randomised Trial | *Bmj Open* | 2014 |
| Di Iacovo F;Moruzzo R;Rossignoli Cm;Scarpellini P | Measuring The Effects Of Transdisciplinary Research: The Case Of A Social Farming Project | *Futures* | 2016 |
| Dias S;Gama A;Simoes D;Mendao L | Implementation Process And Impacts Of A Participatory Hiv Research Project With Key Populations | *Biomed Research International* | 2018 |
| Dickerson J;Bird Pk;Bryant M;Dharni N;Bridges S;Willan K;Ahern S;Dunn A;Nielsen D;Uphoff Ep;Bywater T;Bowyer Crane C;Sahota P;Small N;Howell M;Thornton G;Pickett Ke;Mceachan Rrc;Wright J;Better Start Bradford Better Start Bsbbs | Integrating Research And System-Wide Practice In Public Health: Lessons Learnt From Better Start Bradford | *Bmc Public Health* | 2019 |
| Dietrich T;Rundle Thiele S;Schuster L;Connor J | Co-Designing Social Marketing Programs | *Journal Of Social Marketing* | 2016 |
| Dixon J;Sindall C | Applying Logics Of Change To The Evaluation Of Community-Development In Health Promotion | *Health Promotion International* | 1994 |
| Doheny S;Milbourne P | Modernization And Devolution: Delivering Services For Older People In Rural Areas Of England And Wales | *Social Policy \& Administration* | 2013 |
| Dunston R;Lee A;Boud D;Brodie P;Chiarella M | Co-Production And Health System Reform - From Re-Imagining To Re-Making | *Australian Journal Of Public Administration* | 2009 |
| Edwards D;Anstey S;Kelly D;Hopkinson J | An Innovation In Curriculum Content And Delivery Of Cancer Education Within Undergraduate Nurse Training In The Uk. What Impact Does This Have On The Knowledge, Attitudes And Confidence In Delivering Cancer Care? | *European Journal Of Oncology Nursing* | 2016 |
| Elliott Kej;Stirling Cm;Martin Aj;Robinson Al;Scott Jl | Coproduction For Sustainability: Seeking The Perspectives Of Informal Dementia Carers' On Capacity Building For Community Services | *Journal Of Community Psychology* | 2017 |
| Eriksson Em | Representative Co-Production: Broadening The Scope Of The Public Service Logic | *Public Management Review* | 2019 |
| Eriksson Em;Nordgren L | From One-Sized To Over-Individualized? Service Logic's Value Creation | *Journal Of Health Organization And Management* | 2018 |
| Essen A;Varlander Sw;Liljedal Kt | Co-Production In Chronic Care: Exploitation And Empowerment | *European Journal Of Marketing* | 2016 |
| Evans Ba;Porter A;Snooks H;Burholt V | A Co-Produced Method To Involve Service Users In Research: The Success Model | *Bmc Medical Research Methodology* | 2019 |
| Evans S;Hills S;Orme J | Doing More For Less? Developing Sustainable Systems Of Social Care In The Context Of Climate Change And Public Spending Cuts | *British Journal Of Social Work* | 2012 |
| Ewert B;Evers A | An Ambiguous Concept: On The Meanings Of Co-Production For Health Care Users And User Organizations? | *Voluntas* | 2014 |
| Fang Ml;Woolrych R;Sixsmith J;Canham S;Battersby L;Sixsmith A | Place-Making With Older Persons: Establishing Sense-Of-Place Through Participatory Community Mapping Workshops | *Social Science \& Medicine* | 2016 |
| Farmer J;Carlisle K;Dickson Swift V;Teasdale S;Kenny A;Taylor J;Croker F;Marini K;Gussy M | Applying Social Innovation Theory To Examine How Community Co-Designed Health Services Develop: Using A Case Study Approach And Mixed Methods | *Bmc Health Services Research* | 2018 |
| Farmer J;Taylor J;Stewart E;Kenny A | Citizen Participation In Health Services Co-Production: A Roadmap For Navigating Participation Types And Outcomes | *Australian Journal Of Primary Health* | 2017 |
| Farmer J;Currie M;Kenny A;Munoz Sa | An Exploration Of The Longer-Term Impacts Of Community Participation In Rural Health Services Design | *Social Science \& Medicine* | 2015 |
| Farmer J;Nimegeer A | Community Participation To Design Rural Primary Healthcare Services | *Bmc Health Services Research* | 2014 |
| Farr M;Banks J;Edwards Hb;Northstone K;Bernard E;Salisbury C;Horwood J | Implementing Online Consultations In Primary Care: A Mixed-Method Evaluation Extending Normalisation Process Theory Through Service Co-Production | *Bmj Open* | 2018 |
| Farshchian Ba;Vilarinho T;Mikalsen M | From Episodes To Continuity Of Care: A Study Of A Call Center For Supporting Independent Living | *Computer Supported Cooperative Work-The Journal Of Collaborative Computing* | 2017 |
| Fazey I;Bunse L;Msika J;Pinke M;Preedy K;Evely Ac;Lambert E;Hastings E;Morris S;Reed Ms | Evaluating Knowledge Exchange In Interdisciplinary And Multi-Stakeholder Research | *Global Environmental Change-Human And Policy Dimensions* | 2014 |
| Fenge La;Fannin A;Hicks C | Co-Production In Scholarly Activity: Valuing The Social Capital Of Lay People And Volunteers | *Journal Of Social Work* | 2012 |
| Filipe A;Renedo A;Marston C | The Co-Production Of What? Knowledge, Values, And Social Relations In Health Care | *Plos Biology* | 2017 |
| Fledderus J;Brandsen T;Honingh Me | User Co-Production Of Public Service Delivery: An Uncertainty Approach | *Public Policy And Administration* | 2015 |
| Fledderus J;Brandsen T;Honingh M | Restoring Trust Through The Co-Production Of Public Services: A Theoretical Elaboration | *Public Management Review* | 2014 |
| Flood C;Barlow S;Simpson A;Burls A;Price A;Cartwright M;Brini S;Serv User Carer Grp Advising Res S Sucgars | What Utility Scores Do Mental Health Service Users, Healthcare Professionals And Members Of The General Public Attribute To Different Health States? A Co-Produced Mixed Methods Online Survey | *Plos One* | 2018 |
| Freebairn L;Atkinson Ja;Kelly Pm;Mcdonnell G;Rychetnik L | Decision Makers' Experience Of Participatory Dynamic Simulation Modelling: Methods For Public Health Policy | *Bmc Medical Informatics And Decision Making* | 2018 |
| Freebairn L;Rychetnik L;Atkinson Ja;Kelly P;Mcdonnell G;Roberts N;Whittall C;Redman S | Knowledge Mobilisation For Policy Development: Implementing Systems Approaches Through Participatory Dynamic Simulation Modelling | *Health Research Policy And Systems* | 2017 |
| Gale N;Brown P;Sidhu M | Co-Production In The Epidemiological Clinic: A Decentred Analysis Of The Tensions In Community-Based, Client-Facing Risk Work | *Social Policy \& Administration* | 2019 |
| Gellatly J;Bee P;Kolade A;Hunter D;Gega L;Callender C;Hope H;Abel Km | Developing An Intervention To Improve The Health Related Quality Of Life In Children And Young People With Serious Parental Mental Illness | *Frontiers In Psychiatry* | 2019 |
| Georgiadis A;Duschinsky R;Perez J;Jones Pb;Russo D;Knight C;Soneson E;Dixon Woods M | Coproducing Healthcare Service Improvement For People With Common Mental Health Disorders Including Psychotic Experiences: A Study Protocol Of A Multiperspective Qualitative Study | *Bmj Open* | 2018 |
| Gillard S;Turner K;Neffgen M | Understanding Recovery In The Context Of Lived Experience Of Personality Disorders: A Collaborative, Qualitative Research Study | *Bmc Psychiatry* | 2015 |
| Gillard S;Simons L;Turner K;Lucock M;Edwards C | Patient And Public Involvement In The Coproduction Of Knowledge: Reflection On The Analysis Of Qualitative Data In A Mental Health Study | *Qualitative Health Research* | 2012 |
| Gillespie J;Magee E;White A;Stewart L | Eat, Play, Learn Well-A Novel Approach To Co-Production And Analysis Grid For Environments Linked To Obesity To Engage Local Communities In A Child Healthy Weight Action Plan | *Public Health* | 2019 |
| Gladman Jrf;Conroy Sp;Ranhoff Ah;Gordon Al | New Horizons In The Implementation And Research Of Comprehensive Geriatric Assessment: Knowing, Doing And The `Know-Do' Gap | *Age And Ageing* | 2016 |
| Glimmerveen L;Nies H;Ybema S | Citizens As Active Participants In Integrated Care: Challenging The Field's Dominant Paradigms | *International Journal Of Integrated Care* | 2019 |
| Glimmerveen L;Ybema S;Nies H | Empowering Citizens Or Mining Resources? The Contested Domain Of Citizen Engagement In Professional Care Services | *Social Science \& Medicine* | 2018 |
| Graham Id;Kothari A;Mccutcheon C;Integrated Knowledge Transla Ikt | Moving Knowledge Into Action For More Effective Practice, Programmes And Policy: Protocol For A Research Programme On Integrated Knowledge Translation | *Implementation Science* | 2018 |
| Greco M | Pragmatics Of Explanation: Creative Accountability In The Care Of ``Medically Unexplained Symptoms' | *Sociological Review* | 2017 |
| Greenhalgh T;Fahy N | Research Impact In The Community-Based Health Sciences: An Analysis Of 162 Case Studies From The 2014 Uk Research Excellence Framework | *Bmc Medicine* | 2015 |
| Gremyr I;Elg M;Smith F;Gustavsson S | Exploring The Phase For Highest Impact On Radicality: A Cross-Sectional Study Of Patient Involvement In Quality Improvement In Swedish Healthcare | *Bmj Open* | 2018 |
| Groenen Cjm;Van Duijnhoven Ntl;Kremer Jam;Scheerhagen M;Vandenbussche Fpha;Faber Mj | Shared Agenda Making For Quality Improvement, Towards More Synergy In Maternity Care | *European Journal Of Obstetrics \& Gynecology And Reproductive Biology* | 2017 |
| Groven Ks;Svendby Eb;Rugseth G | Ethical Sensitivity In Co-Production: Openness And Doubt When Young Women Participate In Research | *Health Care For Women International* | 2019 |
| Grundy Ac;Walker L;Meade O;Fraser C;Cree L;Bee P;Lovell K;Callaghan P | Evaluation Of A Co-Delivered Training Package For Community Mental Health Professionals On Service User- And Carer-Involved Care Planning | *Journal Of Psychiatric And Mental Health Nursing* | 2017 |
| Grundy A;Keetharuth Ad;Barber R;Carlton J;Connell J;Buck Et;Barkham M;Ricketts T;Robotham D;Rose D;Kay J;Hanlon R;Brazier J | Public Involvement In Health Outcomes Research: Lessons Learnt From The Development Of The Recovering Quality Of Life (Reqol) Measures | *Health And Quality Of Life Outcomes* | 2019 |
| Guo L;Arnould Ej;Gruen Tw;Tang C | Socializing To Co-Produce: Pathways To Consumers' Financial Well-Being | *Journal Of Service Research* | 2013 |
| Gwyther H;Shaw R;Jaime Dauden Ea;D Avanzo B;Kurpas D;Bujnowska Fedak M;Kujawa T;Marcucci M;Cano A;Holland C | Understanding Frailty: A Qualitative Study Of European Healthcare Policy-Makers' Approaches To Frailty Screening And Management | *Bmj Open* | 2018 |
| Gyi D;Sang K;Haslam C | Participatory Ergonomics: Co-Developing Interventions To Reduce The Risk Of Musculoskeletal Symptoms In Business Drivers | *Ergonomics* | 2013 |
| Hafford Letchfield T;Simpson P;Willis Pb;Almack K | Developing Inclusive Residential Care For Older Lesbian, Gay, Bisexual And Trans (Lgbt) People: An Evaluation Of The Care Home Challenge Action Research Project | *Health \& Social Care In The Community* | 2018 |
| Hales Sa;Fossey J | Caring For Me And You: The Co-Production Of A Computerised Cognitive Behavioural Therapy (Ccbt) Package For Carers Of People With Dementia | *Aging \& Mental Health* | 2018 |
| Hamalainen Rm;Aro Ar;Lau Cj;Rus D;Cori L;Syed Am;Res Into Policy Enhance Phys Reipoep | Cross-Sector Cooperation In Health-Enhancing Physical Activity Policymaking: More Potential Than Achievements? | *Health Research Policy And Systems* | 2016 |
| Happell B;Platania Phung C;Scholz B;Bocking J;Horgan A;Manning F;Doody R;Hals E;Granerud A;Lahti M;Pullo J;Vatula A;Koski J;Van Der Vaart Kj;Allon J;Griffin M;Russell S;Macgabhann L;Bjornsson E;Biering P | Changing Attitudes: The Impact Of Expert By Experience Involvement In Mental Health Nursing Education: An International Survey Study | *International Journal Of Mental Health Nursing* | 2019 |
| Happell B;Gordon S;Bocking J;Ellis P;Roper C;Liggins J;Scholz B;Platania Phung C | ``Chipping Away'': Non-Consumer Researcher Perspectives On Barriers To | *Journal Of Mental Health* | 2019 |
| Happell B;Gordon S;Bocking J;Ellis P;Roper C;Liggins J;Platania Phung C;Scholz B | Mental Health Researchers' Views About Service User Research: A Literature Review | *Issues In Mental Health Nursing* | 2018 |
| Happell B;Scholz B;Gordon S;Bocking J;Ellis P;Roper C;Liggins J;Platania Phung C | ``I Don't Think We've Quite Got There Yet'': The Experience Of | *Journal Of Psychiatric And Mental Health Nursing* | 2018 |
| Happell B;Gordon S;Bocking J;Ellis P;Roper C;Liggins J;Platania Phung C;Scholz B | How Did I Not See That? Perspectives Of Nonconsumer Mental Health Researchers On The Benefits Of Collaborative Research With Consumers | *International Journal Of Mental Health Nursing* | 2018 |
| Happell B;Bennetts W;Tohotoa J;Platania Phung C;Wynaden D | Nothing Without Vision! The Views Of Consumers And Mental Health Nurses About Consumer Involvement In Mental Health Nursing Education | *Collegian* | 2016 |
| Hardyman W;Daunt Kl;Kitchener M | Value Co-Creation Through Patient Engagement In Health Care: A Micro-Level Approach And Research Agenda | *Public Management Review* | 2015 |
| Harvey G;Fitzgerald L;Fielden S;Mcbride A;Waterman H;Bamford D;Kislov R;Boaden R | The Nihr Collaboration For Leadership In Applied Health Research And Care (Clahrc) For Greater Manchester: Combining Empirical, Theoretical And Experiential Evidence To Design And Evaluate A Large-Scale Implementation Strategy | *Implementation Science* | 2011 |
| Hawkins J;Madden K;Fletcher A;Midgley L;Grant A;Cox G;Moore L;Campbell R;Murphy S;Bonell C;White J | Development Of A Framework For The Co-Production And Prototyping Of Public Health Interventions | *Bmc Public Health* | 2017 |
| Heaton J;Day J;Britten N | Collaborative Research And The Co-Production Of Knowledge For Practice: An Illustrative Case Study | *Implementation Science* | 2016 |
| Heaton J;Day J;Britten N | Inside The ``Black Box'' Of A Knowledge Translation Program In Applied | *Qualitative Health Research* | 2015 |
| Hendry M;Pasterfield D;Gollins S;Adams R;Evans M;Fiander A;Robling M;Campbell C;Bekkers Mj;Hiscock J;Nafees S;Rose J;Stanley M;Williams O;Makin M;Wilkinson C | Talking About Human Papillomavirus And Cancer: Development Of Consultation Guides Through Lay And Professional Stakeholder Coproduction Using Qualitative, Quantitative And Secondary Data | *Bmj Open* | 2017 |
| Henwood F;Hart A | Articulating Gender In The Context Of Icts In Health Care: The Case Of Electronic Patient Records In The Maternity Services | *Critical Social Policy* | 2003 |
| Hernandez Ar;Hurtig Ak;Dahlblom K;Sebastian Ms | Integrating Views On Support For Mid-Level Health Worker Performance: A Concept Mapping Study With Regional Health System Actors In Rural Guatemala | *International Journal For Equity In Health* | 2015 |
| Hesselink G;Johnson J;Batalden P;Carlson M;Geense W;Groenewoud S;Jones S;Roy B;Sansone C;Wolf Jrlm;Bart B;Wollersheim H | `Reframing Healthcare Services Through The Lens Of Co-Production' (Rhelaunch): A Study Protocol For A Mixed Methods Evaluation Of Mechanisms By Which Healthcare And Social Services Impact The Health And Well-Being Of Patients With Copd And Chf In The Usa And The Netherlands | *Bmj Open* | 2017 |
| Hewison A;Gale N;Shapiro J | Co-Production In Research: Some Reflections On The Experience Of Engaging Practitioners In Health Research | *Public Money \& Management* | 2012 |
| Hill Jc;Kang S;Benedetto E;Myers H;Blackburn S;Smith S;Dunn Km;Hay E;Rees J;Beard D;Glyn Jones S;Barker K;Ellis B;Fitzpatrick R;Price A | Development And Initial Cohort Validation Of The Arthritis Research Uk Musculoskeletal Health Questionnaire (Msk-Hq) For Use Across Musculoskeletal Care Pathways | *Bmj Open* | 2016 |
| Hoekstra F;Ginis Kam;Allan V;Kothari A;Gainforth Hl | Evaluating The Impact Of A Network Of Research Partnerships: A Longitudinal Multiple Case Study Protocol | *Health Research Policy And Systems* | 2018 |
| Holland Hart Dm;Addis Sm;Edwards A;Kenkre Je;Wood F | Coproduction And Health: Public And Clinicians' Perceptions Of The Barriers And Facilitators | *Health Expectations* | 2019 |
| Holmboe Es;Foster Tc;Ogrinc G | Co-Creating Quality In Health Care Through Learning And Dissemination | *Journal Of Continuing Education In The Health Professions* | 2016 |
| Holmboe Es;Batalden P | Achieving The Desired Transformation: Thoughts On Next Steps For Outcomes-Based Medical Education | *Academic Medicine* | 2015 |
| Hopwood N;Edwards A | How Common Knowledge Is Constructed And Why It Matters In Collaboration Between Professionals And Clients | *International Journal Of Educational Research* | 2017 |
| Horgan A;Manning F;Bocking J;Happell B;Lahti M;Doody R;Griffin M;Bradley Sk;Russell S;Bjornsson E;O Donovan M;Macgabhann L;Savage E;Pulli J;Goodwin J;Van Der Vaart Kj;O Sullivan H;Dorrity C;Ellila H;Allon J;Hals E;Sitvast J;Granerud A;Biering P | ``To Be Treated As A Human': Using Co-Production To Explore Experts By Experience Involvement In Mental Health Nursing Education - The Commune Project | *International Journal Of Mental Health Nursing* | 2018 |
| Howieson B | Mutuality In The Provision Of Scottish Healthcare | *Scottish Medical Journal* | 2015 |
| Hughes M;Duffy C | Public Involvement In Health And Social Sciences Research: A Concept Analysis | *Health Expectations* | 2018 |
| Hyde P;Davies Hto | Service Design, Culture And Performance: Collusion And Co-Production In Health Care | *Human Relations* | 2004 |
| Irvine F;Yeung Eyw;Partridge M;Simcock P | The Impact Of Personalisation On People From Chinese Backgrounds: Qualitative Accounts Of Social Care Experience | *Health \& Social Care In The Community* | 2017 |
| Irving A;Turner J;Marsh M;Broadway Parkinson A;Fall D;Coster J;Siriwardena An | A Coproduced Patient And Public Event: An Approach To Developing And Prioritizing Ambulance Performance Measures | *Health Expectations* | 2018 |
| Isett Kr;Miranda J | Watching Sausage Being Made: Lessons Learned From The Co-Production Of Governance In A Behavioural Health System | *Public Management Review* | 2015 |
| Jaspers S;Steen T | Realizing Public Values: Enhancement Or Obstruction? Exploring Value Tensions And Coping Strategies In The Co-Production Of Social Care | *Public Management Review* | 2019 |
| Jennings H;Slade M;Bates P;Munday E;Toney R | Best Practice Framework For Patient And Public Involvement (Ppi) In Collaborative Data Analysis Of Qualitative Mental Health Research: Methodology Development And Refinement | *Bmc Psychiatry* | 2018 |
| Jo S;Nabatchi T | Coproducing Healthcare: Individual-Level Impacts Of Engaging Citizens To Develop Recommendations For Reducing Diagnostic Error | *Public Management Review* | 2019 |
| Jones F;Postges H;Brimicombe L | Building Bridges Between Healthcare Professionals, Patients And Families: A Coproduced And Integrated Approach To Self-Management Support In Stroke | *Neurorehabilitation* | 2016 |
| Jones M;Ferguson M;Walsh S;Martinez L;Marsh M;Cronin K;Procter N | Perspectives Of Rural Health And Human Service Practitioners Following Suicide Prevention Training Programme In Australia: A Thematic Analysis | *Health \& Social Care In The Community* | 2018 |
| Kaartemo V;Kansakoski H | Information And Knowledge Processes In Health Care Value Co-Creation And Co-Destruction | *Sage Open* | 2018 |
| Kamal Ah;Kirkland Kb;Meier De;Morgan Ts;Nelson Ec;Pantilat Sz | A Person-Centered, Registry-Based Learning Health System For Palliative Care: A Path To Coproducing Better Outcomes, Experience, Value, And Science | *Journal Of Palliative Medicine* | 2018 |
| Kavcic M;Pahor M;Domajnko B | User Involvement In Slovenian Healthcare | *Journal Of Health Organization And Management* | 2015 |
| Khreis H;Warsow Km;Verlinghieri E;Guzman A;Pellecuer L;Ferreira A;Jones I;Heinen E;Rojas Rueda D;Mueller N;Schepers P;Lucas K;Nieuwenhuijsen M | The Health Impacts Of Traffic-Related Exposures In Urban Areas: Understanding Real Effects, Underlying Driving Forces And Co-Producing Future Directions | *Journal Of Transport \& Health* | 2016 |
| Kidd S;Kenny A;Mckinstry C | Exploring The Meaning Of Recovery-Oriented Care: An Action-Research Study | *International Journal Of Mental Health Nursing* | 2015 |
| Kirkegaard S;Andersen D | Co-Production In Community Mental Health Services: Blurred Boundaries Or A Game Of Pretend? | *Sociology Of Health \& Illness* | 2018 |
| Kislov R;Wilson Pm;Knowles S;Boaden R | Learning From The Emergence Of Nihr Collaborations For Leadership In Applied Health Research And Care (Clahrcs): A Systematic Review Of Evaluations | *Implementation Science* | 2018 |
| Knowles S;Hays R;Senra H;Bower P;Locock L;Protheroe J;Sanders C;Daker White G | Empowering People To Help Speak Up About Safety In Primary Care: Using Codesign To Involve Patients And Professionals In Developing New Interventions For Patients With Multimorbidity | *Health Expectations* | 2018 |
| Koivisto J;Anttila H;Ikonen T;Reiman Mottonen P | A Systematic Model For Evaluating The Patient Aspects Of Health Technologies | *Evidence \& Policy* | 2010 |
| Kok Mo;Vaandrager L;Bal R;Schuit J | Practitioner Opinions On Health Promotion Interventions That Work: Opening The `Black Box' Of A Linear Evidence-Based Approach | *Social Science \& Medicine* | 2012 |
| Kothari A;Wathen Cn | Integrated Knowledge Translation: Digging Deeper, Moving Forward | *Journal Of Epidemiology And Community Health* | 2017 |
| Laitinen I;Kinder T;Stenvall J | Street-Level New Public Governances In Integrated Services-As-A-System | *Public Management Review* | 2018 |
| Lambert N;Carr S | ``Outside The Original Remit': Co-Production In Uk Mental Health Research, Lessons From The Field | *International Journal Of Mental Health Nursing* | 2018 |
| Langley J;Wolstenholme D;Cooke J | `Collective Making' As Knowledge Mobilisation: The Contribution Of Participatory Design In The Co-Creation Of Knowledge In Healthcare | *Bmc Health Services Research* | 2018 |
| Latif A;Carter T;Rychwalska Brown L;Wharrad H;Manning J | Co-Producing A Digital Educational Programme For Registered Children's Nurses To Improve Care Of Children And Young People Admitted With Self-Harm | *Journal Of Child Health Care* | 2017 |
| Latif A;Pollock K;Anderson C;Waring J;Solomon J;Chen Lc;Anderson E;Gulzar S;Abbasi N;Wharrad H | Supporting Underserved Patients With Their Medicines: A Study Protocol For A Patient/Professional Coproduced Education Intervention For Community Pharmacy Staff To Improve The Provision And Delivery Of Medicine Use Reviews (Murs) | *Bmj Open* | 2016 |
| Learmonth Am;Henderson Ej;Hunter Dj | Securing Systems Leadership By Local Government Through Health And Wellbeing Strategies | *Journal Of Public Health* | 2018 |
| Ledger A;Slade B | Coproduction Without Experts: A Study Of People Involved In Community Health And Well-Being Service Delivery | *Studies In Continuing Education* | 2015 |
| Lehmann U;Gilson L | Action Learning For Health System Governance: The Reward And Challenge Of Co-Production | *Health Policy And Planning* | 2015 |
| Lembani M;Teddy G;Molosiwa D;Hwabamungu B | Post-Doctoral Research Fellowship As A Health Policy And Systems Research Capacity Development Intervention: A Case Of The Chesai Initiative | *Health Research Policy And Systems* | 2016 |
| Leonard K;Hafford Letchfield T;Couchman W | `We're All Going Bali': Utilising Gamelan As An Educational Resource For Leadership And Teamwork In Post-Qualifying Education In Health And Social Care | *British Journal Of Social Work* | 2013 |
| Lichon M;Kavcic M;Masterson D | A Comparative Study Of Contemporary User Involvement Within Healthcare Systems Across England, Poland And Slovenia | *Journal Of Health Organization And Management* | 2015 |
| Lim E;Wynaden D;Heslop K | Recovery-Focussed Care: How It Can Be Utilized To Reduce Aggression In The Acute Mental Health Setting | *International Journal Of Mental Health Nursing* | 2017 |
| Lucas Pj;Allnock D;Jessiman T | How Are European Birth-Cohort Studies Engaging And Consulting With Young Cohort Members? | *Bmc Medical Research Methodology* | 2013 |
| Lundin M;Makitalo A | Co-Designing Technologies In The Context Of Hypertension Care: Negotiating Participation And Technology Use In Design Meetings | *Informatics For Health \& Social Care* | 2017 |
| Lwembe S;Green Sa;Chigwende J;Ojwang T;Dennis R | Co-Production As An Approach To Developing Stakeholder Partnerships To Reduce Mental Health Inequalities: An Evaluation Of A Pilot Service | *Primary Health Care Research And Development* | 2017 |
| Mackintosh N;Sandall J;Collison C;Carter W;Harris J | Employing The Arts For Knowledge Production And Translation: Visualizing New Possibilities For Women Speaking Up About Safety Concerns In Maternity | *Health Expectations* | 2018 |
| Malden S;Hughes Ar;Gibson Am;Bardid F;Androutsos O;De Craemer M;Manios Y;Summerbell C;Cardon G;Reilly Jj | Adapting The Toybox Obesity Prevention Intervention For Use In Scottish Preschools: Protocol For A Feasibility Cluster Randomised Controlled Trial | *Bmj Open* | 2018 |
| Manikam L;Shah R;Reed K;Santini G;Lakhanpaul M | Using A Co-Production Prioritization Exercise Involving South Asian Children, Young People And Their Families To Identify Health Priorities Requiring Further Research And Public Awareness | *Health Expectations* | 2017 |
| Marston C;Hinton R;Kean S;Baral S;Ahuja A;Costello A;Portela A | Community Participation For Transformative Action On Women's, Children's And Adolescents' Health | *Bulletin Of The World Health Organization* | 2016 |
| Martin D | Lean In A Cold Fiscal Climate: The Public Sector In An Age Of Reduced Resources | *Public Money \& Management* | 2018 |
| Mayer C;Mckenzie K | `...It Shows That There's No Limits': The Psychological Impact Of Co-Production For Experts By Experience Working In Youth Mental Health | *Health \& Social Care In The Community* | 2017 |
| Mcallister Jw;Keehn Rmn;Rodgers R;Lock Tm | Care Coordination Using A Shared Plan Of Care Approach: From Model To Practice | *Journal Of Pediatric Nursing-Nursing Care Of Children \& Families* | 2018 |
| Mccoll Kennedy Jr;Vargo Sl;Dagger Ts;Sweeney Jc;Van Kasteren Y | Health Care Customer Value Cocreation Practice Styles | *Journal Of Service Research* | 2012 |
| Mcintosh Gl | Exploration Of The Perceived Impact Of Carer Involvement In Mental Health Nurse Education: Values, Attitudes And Making A Difference | *Nurse Education In Practice* | 2018 |
| Meer T;Muller A | ``They Treat Us Like We're Not There'': Queer Bodies And The Social | *Health \& Place* | 2017 |
| Mende M;Scott Ml;Bitner Mj;Ostrom Al | Activating Consumers For Better Service Coproduction Outcomes Through Eustress: The Interplay Of Firm-Assigned Workload, Service Literacy, And Organizational Support | *Journal Of Public Policy \& Marketing* | 2017 |
| Mifsud M;Cases As;N Goala G | Service Appropriation: How Do Customers Make The Service Their Own? | *Journal Of Service Management* | 2015 |
| Montenegro M;Greenhill B | Evaluating `Freda Challenge': A Coproduced Human Rights Board Game In Services For People With Intellectual Disabilities | *Journal Of Applied Research In Intellectual Disabilities* | 2015 |
| Montgomery Cm | Making Prevention Public: The Co-Production Of Gender And Technology In Hiv Prevention Research | *Social Studies Of Science* | 2012 |
| Morris Zs;Bullock A;Atwell C | Developing Engagement, Linkage And Exchange Between Health Services Managers And Researchers: Experience From The Uk | *Journal Of Health Services Research \& Policy* | 2013 |
| Mort M;Roberts C;Callen B | Ageing With Telecare: Care Or Coercion In Austerity? | *Sociology Of Health \& Illness* | 2013 |
| Morton M;Paice E | Co-Production At The Strategic Level: Co-Designing An Integrated Care System With Lay Partners In North West London, England | *International Journal Of Integrated Care* | 2016 |
| Nambiar D;Dasgupta R;Sundararaman T;Ganesan P;Gupta S | Reflections On Participation And Knowledge-Making As Part Of India's National Urban Health Mission Technical Resource Group Recommendation Exercise | *International Journal Of Health Services* | 2018 |
| Neech Sgb;Scott H;Priest Hm;Bradley Ej;Tweed Ae | Experiences Of User Involvement In Mental Health Settings: User Motivations And Benefits | *Journal Of Psychiatric And Mental Health Nursing* | 2018 |
| Newman Taylor K;Garner C;Vernon Wilson E;Paas Khw;Herbert L;Au Yeung Sk | Psychometric Evaluation Of The Hope, Agency And Opportunity (Hao), A Brief Measure Of Mental Health Recovery | *Journal Of Mental Health* | 2017 |
| Newman Taylor K;Stone N;Valentine P;Hooks Z;Sault K | The Recovery College: A Unique Service Approach And Qualitative Evaluation | *Psychiatric Rehabilitation Journal* | 2016 |
| Nimegeer A;Farmer J;West C;Currie M | Addressing The Problem Of Rural Community Engagement In Healthcare Service Design | *Health \& Place* | 2011 |
| Nudurupati Ss;Bhattacharya A;Lascelles D;Caton N | Strategic Sourcing With Multi-Stakeholders Through Value Co-Creation: An Evidence From Global Health Care Company | *International Journal Of Production Economics* | 2015 |
| Nunn Js;Tiller J;Fransquet P;Lacaze P | Public Involvement In Global Genomics Research: A Scoping Review | *Frontiers In Public Health* | 2019 |
| Nuti S;Noto G;Vola F;Vainieri M | Let's Play The Patients Music: A New Generation Of Performance Measurement Systems In Healthcare | *Management Decision* | 2018 |
| Nystrom Me;Karltun J;Keller C;Gare Ba | Collaborative And Partnership Research For Improvement Of Health And Social Services: Researcher's Experiences From 20 Projects | *Health Research Policy And Systems* | 2018 |
| O Brien Ma;Carson A;Barbera L;Brouwers Mc;Earle Cc;Graham Id;Mittmann N;Grunfeld E | Variable Participation Of Knowledge Users In Cancer Health Services Research: Results Of A Multiple Case Study | *Bmc Medical Research Methodology* | 2018 |
| Oliver K;Kothari A;Mays N | The Dark Side Of Coproduction: Do The Costs Outweigh The Benefits For Health Research? | *Health Research Policy And Systems* | 2019 |
| Ongolo Zogo P;Lavis Jn;Tomson G;Sewankambo Nk | Assessing The Influence Of Knowledge Translation Platforms On Health System Policy Processes To Achieve The Health Millennium Development Goals In Cameroon And Uganda: A Comparative Case Study | *Health Policy And Planning* | 2018 |
| Osborne Sp;Radnor Z;Strokosch K | Co-Production And The Co-Creation Of Value In Public Services A Suitable Case For Treatment? | *Public Management Review* | 2016 |
| Ottmann G;Laragy C;Allen J;Feldman P | Coproduction In Practice: Participatory Action Research To Develop A Model Of Community Aged Care | *Systemic Practice And Action Research* | 2011 |
| Owens J;Cribb A | Conflict In Medical Co-Production: Can A Stratified Conception Of Health Help? | *Health Care Analysis* | 2012 |
| Page R;Shankar R;Mclean Bn;Hanna J;Newman C | Digital Care In Epilepsy: A Conceptual Framework For Technological Therapies | *Frontiers In Neurology* | 2018 |
| Palumbo R;Manna R | What If Things Go Wrong In Co-Producing Health Services? Exploring The Implementation Problems Of Health Care Co-Production | *Policy And Society* | 2018 |
| Palumbo R;Annarumma C;Adinolfi P;Musella M | The Missing Link To Patient Engagement In Italy The Role Of Health Literacy In Enabling Patients | *Journal Of Health Organization And Management* | 2016 |
| Parrado S;Van Ryzin Gg;Bovaird T;Loeffler E | Correlates Of Co-Production: Evidence From A Five-Nation Survey Of Citizens | *International Public Management Journal* | 2013 |
| Pavolini E;Spina E | Users' Involvement In The Italian Nhs: The Role Of Associations And Self-Help Groups | *Journal Of Health Organization And Management* | 2015 |
| Pearson C;Watson N;Manji K | Changing The Culture Of Social Care In Scotland: Has A Shift To Personalization Brought About Transformative Change? | *Social Policy \& Administration* | 2018 |
| Petrakis M;Brophy L;Lewis J;Stylianou M;Scott M;Cocks N;Buckley L;Halloran K | Consumer Measures And Research Co- Production: A Pilot Study Evaluating The Recovery Orientation Of A Mental Health Program Collaboration | *Asia Pacific Journal Of Social Work And Development* | 2014 |
| Pors As | Digital Displacements In Patient-Professional Relations: Four Modes Of Organizational Patient Involvement | *Journal Of Health Organization And Management* | 2018 |
| Procter R;Wherton J;Greenhalgh T | Hidden Work And The Challenges Of Scalability And Sustainability In Ambulatory Assisted Living | *Acm Transactions On Computer-Human Interaction* | 2018 |
| Procter R;Wherton J;Greenhalgh T;Sugarhood P;Rouncefield M;Hinder S | Telecare Call Centre Work And Ageing In Place | *Computer Supported Cooperative Work-The Journal Of Collaborative Computing* | 2016 |
| Procter R;Greenhalgh T;Wherton J;Sugarhood P;Rouncefield M;Hinder S | The Day-To-Day Co-Production Of Ageing In Place | *Computer Supported Cooperative Work-The Journal Of Collaborative Computing And Work Practices* | 2014 |
| Radl Karimi C;Nicolaisen A;Sodemann M;Batalden P;Von Plessen C | Coproduction Of Healthcare Service With Immigrant Patients: Protocol Of A Scoping Review | *Bmj Open* | 2018 |
| Rae S | Service Users Perspectives In Promise And Research | *Psychiatria Danubina* | 2017 |
| Raffay J;Wood E;Todd A | Service User Views Of Spiritual And Pastoral Care (Chaplaincy) In Nhs Mental Health Services: A Co-Produced Constructivist Grounded Theory Investigation | *Bmc Psychiatry* | 2016 |
| Ramanadhan S;Davis Mm;Armstrong R;Baquero B;Ko Lk;Leng Jc;Salloum Rg;Vaughn Na;Brownson Rc | Participatory Implementation Science To Increase The Impact Of Evidence-Based Cancer Prevention And Control | *Cancer Causes \& Control* | 2018 |
| Rantamaki Nj | Co-Production In The Context Of Finnish Social Services And Health Care: A Challenge And A Possibility For A New Kind Of Democracy | *Voluntas* | 2017 |
| Rapaport P;Webster L;Horsley R;Kyle Sd;Kinnunen Km;Hallam B;Pickett J;Cooper C;Espie Ca;Livingston G | An Intervention To Improve Sleep For People Living With Dementia: Reflections On The Development And Co-Production Of Dreams:Start (Dementia Related Manual For Sleep: Strategies For Relatives) | *Dementia-International Journal Of Social Research And Practice* | 2018 |
| Realpe Ax;Wallace Lm;Adams Ae;Kidd Jm | The Development Of A Prototype Measure Of The Co-Production Of Health In Routine Consultations For People With Long-Term Conditions | *Patient Education And Counseling* | 2015 |
| Redwood S;Brangan E;Leach V;Horwood J;Donovan Jl | Integration Of Research And Practice To Improve Public Health And Healthcare Delivery Through A Collaborative `Health Integration Team' Model - A Qualitative Investigation | *Bmc Health Services Research* | 2016 |
| Reeve J;Cooper L;Harrington S;Rosbottom P;Watkins J | Developing, Delivering And Evaluating Primary Mental Health Care: The Co-Production Of A New Complex Intervention | *Bmc Health Services Research* | 2016 |
| Reeves P;Deeming S;Ramanathan S;Wiggers J;Wolfenden L;Searles A | Measurement Of The Translation And Impact From A Childhood Obesity Trial Programme: Rationale And Protocol For A Research Impact Assessment | *Health Research Policy And Systems* | 2017 |
| Rhodes P;Mcdonald R;Campbell S;Daker White G;Sanders C | Sensemaking And The Co-Production Of Safety: A Qualitative Study Of Primary Medical Care Patients | *Sociology Of Health \& Illness* | 2016 |
| Roberts A;Greenhill B;Talbot A;Cuzak M | Standing Up For My Human Rights': A Group's Journey Beyond Consultation Towards Co-Production | *British Journal Of Learning Disabilities* | 2012 |
| Robinson T;Skouteris H;Melder A;Bailey C;Morris H;Garad R;Teede Hj | Application Of Monash Centre For Health Research And Implementation Framework To The Development Of Polycycstic Ovary Syndrome Guideline: A Case Study On Implementation | *Seminars In Reproductive Medicine* | 2018 |
| Rowley E;Morriss R;Currie G;Schneider J | Research Into Practice: Collaboration For Leadership In Applied Health Research And Care (Clahrc) For Nottinghamshire, Derbyshire, Lincolnshire (Ndl) | *Implementation Science* | 2012 |
| Ruetten A;Frahsa A;Abel T;Bergmann M;De Leeuw E;Hunter D;Jansen M;King A;Potvin L | Co-Producing Active Lifestyles As Whole-System-Approach: Theory, Intervention And Knowledge-To-Action Implications | *Health Promotion International* | 2019 |
| Rutkowski K;Rahman Y;Halter M | Development And Feasibility Of The Use Of An Assessment Tool Measuring Treatment Efficacy In Patients With Trimethylaminuria: A Mixed Methods Study | *Journal Of Inherited Metabolic Disease* | 2019 |
| Rycroft Malone J;Burton Cr;Wilkinson J;Harvey G;Mccormack B;Baker R;Dopson S;Graham Id;Staniszewska S;Thompson C;Ariss S;Melville Richards L;Williams L | Collective Action For Implementation: A Realist Evaluation Of Organisational Collaboration In Healthcare | *Implementation Science* | 2016 |
| Sabadosa Ka;Batalden Pb | The Interdependent Roles Of Patients, Families And Professionals In Cystic Fibrosis: A System For The Coproduction Of Healthcare And Its Improvement | *Bmj Quality \& Safety* | 2014 |
| Sadler E;Porat T;Marshall I;Hoang U;Curcin V;Wolfe Cda;Mckevitt C | Shaping Innovations In Long-Term Care For Stroke Survivors With Multimorbidity Through Stakeholder Engagement | *Plos One* | 2017 |
| Samorinha C;Lichon M;Silva S;Dent M | User Involvement In Assisted Reproductive Technologies: England And Portugal | *Journal Of Health Organization And Management* | 2015 |
| Sawtell M;Sweeney L;Wiggins M;Salisbury C;Eldridge S;Greenberg L;Hunter R;Kaur I;Mccourt C;Hatherall B;Findlay G;Morris J;Reading S;Renton A;Adekoya R;Green B;Harvey B;Latham S;Patel K;Vanlessen L;Harden A | Evaluation Of Community-Level Interventions To Increase Early Initiation Of Antenatal Care In Pregnancy: Protocol For The Community Reach Study, A Cluster Randomised Controlled Trial With Integrated Process And Economic Evaluations | *Trials* | 2018 |
| Sharma S;Conduit J | Cocreation Culture In Health Care Organizations | *Journal Of Service Research* | 2016 |
| Silarova B;Nelis Sm;Ashworth Rm;Ballard C;Bienkiewicz M;Henderson C;Hillman A;Hindle Jv;Hughes Jc;Lamont Ra;Litherland R;Jones Ir;Jones Rw;Knapp M;Kotting P;Martyr A;Matthews Fe;Morris Rg;Quinn C;Regan J;Rusted Jm;Van Den Heuvel Ea;Victor Cr;Wu Yt;Clare L | Protocol For The Ideal-2 Longitudinal Study: Following The Experiences Of People With Dementia And Their Primary Carers To Understand What Contributes To Living Well With Dementia And Enhances Active Life | *Bmc Public Health* | 2018 |
| Skalen P;Karlsson J;Engen M;Magnusson Pr | Understanding Public Service Innovation As Resource Integration And Creation Of Value Propositions | *Australian Journal Of Public Administration* | 2018 |
| Smith S;Ward V | The Role Of Boundary Maintenance And Blurring In A Uk Collaborative Research Project: How Researchers And Health Service Managers Made Sense Of New Ways Of Working | *Social Science \& Medicine* | 2015 |
| Sorrentino M;Guglielmetti C;Gilardi S;Marsilio M | Health Care Services And The Coproduction Puzzle: Filling In The Blanks | *Administration \& Society* | 2017 |
| Soto C;Strain Wd | Tackling Clinical Inertia: Use Of Coproduction To Improve Patient Engagement | *Journal Of Diabetes* | 2018 |
| Spanjol J;Cui As;Nakata C;Sharp Lk;Crawford Sy;Xiao Y;Watson Manheim Mb | Co-Production Of Prolonged, Complex, And Negative Services: An Examination Of Medication Adherence In Chronically Ill Individuals | *Journal Of Service Research* | 2015 |
| Staniszewska S;Denegri S;Matthews R;Minogue V | Reviewing Progress In Public Involvement In Nihr Research: Developing And Implementing A New Vision For The Future | *Bmj Open* | 2018 |
| Stomski Nj;Morrison P | Participation In Mental Healthcare: A Qualitative Meta-Synthesis | *International Journal Of Mental Health Systems* | 2017 |
| Sutton E;Dixon Woods M;Tarrant C | Ethnographic Process Evaluation Of A Quality Improvement Project To Improve Transitions Of Care For Older People | *Bmj Open* | 2016 |
| Taylor Phillips S;Clarke A;Grove A;Swan J;Parsons H;Gkeredakis E;Mills P;Powell J;Nicolini D;Roginski C;Scarbrough H | Coproduction In Commissioning Decisions: Is There An Association With Decision Satisfaction For Commissioners Working In The Nhs? A Cross-Sectional Survey 2010/2011 | *Bmj Open* | 2014 |
| Thom K;Burnside D | Sharing Power In Criminal Justice: The Potential Of Co-Production For Offenders Experiencing Mental Health And Addictions In New Zealand | *International Journal Of Mental Health Nursing* | 2018 |
| Thomsen Mk;Jakobsen M | Influencing Citizen Coproduction By Sending Encouragement And Advice: A Field Experiment | *International Public Management Journal* | 2015 |
| Thorne K;Andersson Gare B;Hult H;Abrandt Dahlgren M | Co-Producing Interprofessional Round Work: Designing Spaces For Patient Partnership | *Quality Management In Health Care* | 2017 |
| Tien Jm;Goldschmidt Clermont Pj | Healthcare: A Complex Service System | *Journal Of Systems Science And Systems Engineering* | 2009 |
| Toney R;Elton D;Munday E;Hamill K;Crowther A;Meddings S;Taylor A;Henderson C;Jennings H;Waring J;Pollock K;Bates P;Slade M | Mechanisms Of Action And Outcomes For Students In Recovery Colleges | *Psychiatric Services* | 2018 |
| Trummer Uf;Mueller Uo;Nowak P;Stidl T;Pelikan Jm | Does Physician-Patient Communication That Aims At Empowering Patients Improve Clinical Outcome? A Case Study | *Patient Education And Counseling* | 2006 |
| Tudisca V;Valente A;Castellani T;Stahl T;Sandu P;Dulf D;Spitters H;Van De Goor I;Radl Karimi C;Syed Ma;Loncarevic N;Lau Cj;Roelofs S;Bertram M;Edwards N;Aro Ar;Repopa Consortium Repopac | Development Of Measurable Indicators To Enhance Public Health Evidence-Informed Policy-Making | *Health Research Policy And Systems* | 2018 |
| Tupasela A | Re-Examining Medical Modernization: Framing The Public In Finnish Biomedical Research Policy | *Public Understanding Of Science* | 2007 |
| Tuurnas Sp;Stenvall J;Rannisto Ph;Harisalo R;Hakari K | Coordinating Co-Production In Complex Network Settings | *European Journal Of Social Work* | 2015 |
| Unwin Pf;Rooney Jm;Osborne N;Cole C | Are Perceptions Of Disability Changed By Involving Service Users And Carers In Qualifying Health And Social Work Training? | *Disability \& Society* | 2017 |
| Vaeggemose U;Ankersen Pv;Aagaard J;Burau V | Co-Production Of Community Mental Health Services: Organising The Interplay Between Public Services And Civil Society In Denmark | *Health \& Social Care In The Community* | 2018 |
| Van De Goor I;Hamalainen Rm;Syed A;Lau Cj;Sandu P;Spitters H;Karlsson Le;Dulf D;Valente A;Castellani T;Aro Ar;Repopa Consortium Repopa | Determinants Of Evidence Use In Public Health Policy Making: Results From A Study Across Six Eu Countries | *Health Policy* | 2017 |
| Van Der Graaf P;Forrest Lf;Adams J;Shucksmith J;White M | How Do Public Health Professionals View And Engage With Research? A Qualitative Interview Study And Stakeholder Workshop Engaging Public Health Professionals And Researchers | *Bmc Public Health* | 2017 |
| Van Eijk Cja;Steen Tps | Why People Co-Produce: Analysing Citizens' Perceptions On Co-Planning Engagement In Health Care Services | *Public Management Review* | 2014 |
| Van Eijk C;Steen T | Why Engage In Co-Production Of Public Services? Mixing Theory And Empirical Evidence | *International Review Of Administrative Sciences* | 2016 |
| Vennik Fd;Van De Bovenkamp Hm;Putters K;Grit Kj | Co-Production In Healthcare: Rhetoric And Practice | *International Review Of Administrative Sciences* | 2016 |
| Vicari S;Cappai F | Health Activism And The Logic Of Connective Action. A Case Study Of Rare Disease Patient Organisations | *Information Communication \& Society* | 2016 |
| Vickers I;Lyon F;Sepulveda L;Mcmullin C | Public Service Innovation And Multiple Institutional Logics: The Case Of Hybrid Social Enterprise Providers Of Health And Wellbeing | *Research Policy* | 2017 |
| Vijn Tw;Wollersheim H;Faber Mj;Fluit Crmg;Kremer Jam | Building A Patient-Centered And Interprofessional Training Program With Patients, Students And Care Professionals: Study Protocol Of A Participatory Design And Evaluation Study | *Bmc Health Services Research* | 2018 |
| Vindrola Padros C;Eyre L;Baxter H;Cramer H;George B;Wye L;Fulop Nj;Utley M;Phillips N;Brindle P;Marshall M | Addressing The Challenges Of Knowledge Co-Production In Quality Improvement: Learning From The Implementation Of The Researcher-In-Residence Model | *Bmj Quality \& Safety* | 2019 |
| Vogus Tj;Mcclelland Le | When The Customer Is The Patient: Lessons From Healthcare Research On Patient Satisfaction And Service Quality Ratings | *Human Resource Management Review* | 2016 |
| Von Peter S;Schulz G | ``I-As-We' - Powerful Boundaries Within The Field Of Mental Health Coproduction | *International Journal Of Mental Health Nursing* | 2018 |
| Voorberg Wh;Bekkers Vjjm;Tummers Lg | A Systematic Review Of Co-Creation And Co-Production: Embarking On The Social Innovation Journey | *Public Management Review* | 2015 |
| Vrangbaek K;Scheele Ce;Kriegbaum M | Voluntary Associations And Co-Production Of Health Promoting Activities For Older Adults: Experiences And Policy Lessons From Denmark | *Health Policy* | 2018 |
| Vrangbaek K | Patient Involvement In Danish Health Care | *Journal Of Health Organization And Management* | 2015 |
| Wale M;Kibsey P;Young L;Dobbyn B;Archer J | New Approaches To Infection Prevention And Control: Implementing A Risk-Based Model Regionally | *International Journal For Quality In Health Care* | 2016 |
| Walsh M;Kittler Mg;Mahal D | Towards A New Paradigm Of Healthcare: Addressing Challenges To Professional Identities Through Community Operational Research | *European Journal Of Operational Research* | 2018 |
| Ward Me;De Brun A;Beirne D;Conway C;Cunningham U;English A;Fitzsimons J;Furlong E;Kane Y;Kelly A;Mcdonnell S;Mcginley S;Monaghan B;Myler A;Nolan E;O Donovan R;O Shea M;Shuhaiber A;Mcauliffe E | Using Co-Design To Develop A Collective Leadership Intervention For Healthcare Teams To Improve Safety Culture | *International Journal Of Environmental Research And Public Health* | 2018 |
| Wehrens R | Beyond Two Communities - From Research Utilization And Knowledge Translation To Co-Production? | *Public Health* | 2014 |
| Weinberg Db;Lusenhop Rw;Gittell Jh;Kautz Cm | Coordination Between Formal Providers And Informal Caregivers | *Health Care Management Review* | 2007 |
| Wherton J;Sugarhood P;Procter R;Hinder S;Greenhalgh T | Co-Production In Practice: How People With Assisted Living Needs Can Help Design And Evolve Technologies And Services | *Implementation Science* | 2015 |
| Wherton J;Sugarhood P;Procter R;Rouncefield M;Dewsbury G;Hinder S;Greenhalgh T | Designing Assisted Living Technologies `In The Wild': Preliminary Experiences With Cultural Probe Methodology | *Bmc Medical Research Methodology* | 2012 |
| Wilberforce M;Glendinning C;Challis D;Fernandez Jl;Jacobs S;Jones K;Knapp M;Manthorpe J;Moran N;Netten A;Stevens M | Implementing Consumer Choice In Long-Term Care: The Impact Of Individual Budgets On Social Care Providers In England | *Social Policy \& Administration* | 2011 |
| Wilde A | Trust, Uncertainty And Identity In Health-Related Decision-Making: The Role Of Key Professionals | *Disability \& Society* | 2014 |
| Williams A;Moore Sc;Shovelton C;Moore L;Murphy S | Process Evaluation Of An Environmental Health Risk Audit And Action Plan Intervention To Reduce Alcohol Related Violence In Licensed Premises | *Bmc Public Health* | 2016 |
| Williams Bn;Kang Sc;Johnson J | (Co)Contamination As The Dark Side Of Co-Production Public Value Failures In Co-Production Processes | *Public Management Review* | 2016 |
| Williamson A;Tait H;El Jardali F;Wolfenden L;Thackway S;Stewart J;O Leary L;Dixon J | How Are Evidence Generation Partnerships Between Researchers And Policy-Makers Enacted In Practice? A Qualitative Interview Study | *Health Research Policy And Systems* | 2019 |
| Willis P;Almack K;Hafford Letchfield T;Simpson P;Billings B;Mall N | Turning The Co-Production Corner: Methodological Reflections From An Action Research Project To Promote Lgbt Inclusion In Care Homes For Older People | *International Journal Of Environmental Research And Public Health* | 2018 |
| Wilson R;Maniatopoulos G;Martin M;Mcloughlin I | Innovating Relationships Taking A Co-Productive Approach To The Shaping Of Telecare Services For Older People | *Information Communication \& Society* | 2012 |
| Windrum P | Third Sector Organizations And The Co-Production Of Health Innovations | *Management Decision* | 2014 |
| Wolfson M;Wagoner Kg;Rhodes Sd;Egan Kl;Sparks M;Ellerbee D;Song Ey;Debinski B;Terrillion A;Vining J;Yang E | Coproduction Of Research Questions And Research Evidence In Public Health: The Study To Prevent Teen Drinking Parties | *Biomed Research International* | 2017 |
| Wu Hyj | The Moral Career Of `Outmates': Towards A History Of Manufactured Mental Disorders In Post-Socialist China | *Medical History* | 2016 |
| Wutzke S;Rowbotham S;Haynes A;Hawe P;Kelly P;Redman S;Davidson S;Stephenson J;Overs M;Wilson A | Knowledge Mobilisation For Chronic Disease Prevention: The Case Of The Australian Prevention Partnership Centre | *Health Research Policy And Systems* | 2018 |
| Yaraghi N;Du Ay;Sharman R;Gopal Rd;Ramesh R | Health Information Exchange As A Multisided Platform: Adoption, Usage, And Practice Involvement In Service Co-Production | *Information Systems Research* | 2015 |
| Yokota F;Biyani M;Islam R;Ahmed A;Nishikitani M;Kikuchi K;Nohara Y;Nakashima N | Lessons Learned From Co-Design And Co-Production In A Portable Health Clinic Research Project In Jaipur District, India (2016-2018) | *Sustainability* | 2018 |
| Yuan Cw;Hanrahan Bv;Rosson Mb;Carroll Jm | Coming Of Old Age: Understanding Older Adults' Engagement And Needs In Coproduction Activities For Healthy Ageing | *Behaviour \& Information Technology* | 2018 |
| Zanetti Ca;Taylor N | Value Co-Creation In Healthcare Through Positive Deviance | *Healthcare-The Journal Of Delivery Science And Innovation* | 2016 |
| Zlotowitz S;Barker C;Moloney O;Howard C | Service Users As The Key To Service Change? Thedevelopment Of An Innovative Intervention Forexcluded Young People | *Child And Adolescent Mental Health* | 2016 |
