# Supplementary material for: Co-production in health policy and management: a comprehensive bibliometric review
Source: BMC Health Serv Res. 2020 Jun 5;20:504. doi: 10.1186/s12913-020-05241-2 (PMC7275357; doi:10.1186/s12913-020-05241-2)
Supplement: Supplementary file 2 — Additional file 2. Description of metadata per performed analysis [file 12913_2020_5241_MOESM2_ESM.docx]

| **Dataset** | **Metadata** | **Description** | **Performed Analysis** | | | |
| --- | --- | --- | --- | --- | --- | --- |
|  |  |  | *Performance analysis* | *Collaboration analysis* | *Co-citation analysis* | *Co-word analysis* |
| 295 Articles | Authors | All co-authors for each document | X | X *(Figure 5)** |  |  |
|  | First Author | First author of each document | X |  |  |  |
|  | N. of Authors per Paper | Number of authors per document | X  *(Table 1)* |  |  |  |
|  | N. of Authors | Total number of authors | X  *(Table 1)* |  |  |  |
|  | Years | Publication year of each document | X *(Figure 2)* |  |  |  |
|  | Sources | Journal where each document was published | X *(Table 2)* |  |  |  |
|  | Authors' country | Affiliation country of all co-authors for each document | X *(Figure 3)* | X *(Figure 4)** |  |  |
|  | Corresponding authors' country | Affiliation country of corresponding author |  | X  *(Table 5)* |  |  |
|  | Total Citation | Number of times each document has been cited | X *(Table 3)** |  |  |  |
|  | Total Citation per year | Yearly average number of times each document has been cited | X *(Table 3)** |  |  |  |
|  | Authors' Keywords | Author's Keywords for each document |  |  |  | X *(Figure 7)** |
|  | References | Cited References in each document | X *(Table 4)** |  | X *(Figure 6)** |  |

***Additional file 2: Description of metadata per performed analysis***

* The quantitative bibliometric analyses have been integrated with the reading of abstracts or full-texts.
