# Supplementary figures and images for: Co-production in health policy and management: a comprehensive bibliometric review
Source: BMC Health Serv Res. 2020 Jun 5;20:504. doi: 10.1186/s12913-020-05241-2 (PMC7275357; doi:10.1186/s12913-020-05241-2)

***Additional file 3: Most productive authors***


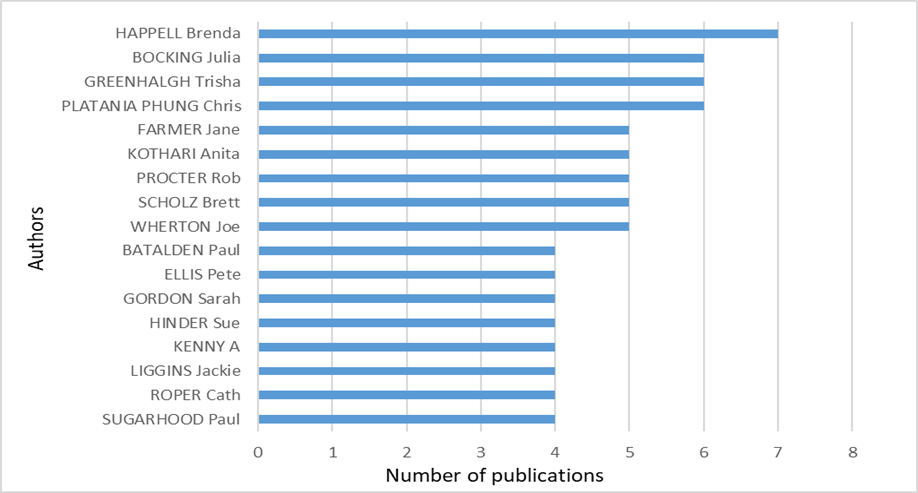

Supplement: Supplementary file 3 — Additional file 3. Most productive authors [file 12913_2020_5241_MOESM3_ESM.docx]
