# Supplementary material for: Co-production in health policy and management: a comprehensive bibliometric review
Source: BMC Health Serv Res. 2020 Jun 5;20:504. doi: 10.1186/s12913-020-05241-2 (PMC7275357; doi:10.1186/s12913-020-05241-2)
Supplement: Supplementary file 4 — Additional file 4. List of authors’ collaboration [file 12913_2020_5241_MOESM4_ESM.docx]

***Additional file 4: List of authors’ collaboration clusters***

| **Cluster** | **Node** | **Affiliation** | **Country** | **Papers** |
| --- | --- | --- | --- | --- |
| 1 | HAPPELL B. | University of Newcastle, Australia | Australia | - Happell, B., Platania‐Phung, C., Scholz, B., Bocking, J., Horgan, A., Manning, F., ... & Pullo, J. (2019). Changing attitudes: The impact of Expert by Experience involvement in Mental Health Nursing Education: An international survey study. *International journal of mental health nursing*, 28(2), 480-491. - Happell, B., Gordon, S., Bocking, J., Ellis, P., Roper, C., Liggins, J., ... & Platania-Phung, C. (2019). “Chipping away”: non-consumer researcher perspectives on barriers to collaborating with consumers in mental health research*. Journal of Mental Health*, 28(1), 49-55. - Happell, B., Gordon, S., Bocking, J., Ellis, P., Roper, C., Liggins, J., ... & Scholz, B. (2018). Mental Health Researchers’ Views About Service User Research: A Literature Review. *Issues in mental health nursing*, 39(12), 1010-1016. - Happell, B., Scholz, B., Gordon, S., Bocking, J., Ellis, P., Roper, C., ... & Platania‐Phung, C. (2018). “I don't think we've quite got there yet”: The experience of allyship for mental health consumer researchers. *Journal of psychiatric and mental health nursing*, 25(8), 453-462. - Happell, B., Gordon, S., Bocking, J., Ellis, P., Roper, C., Liggins, J., ... & Scholz, B. (2018). How did I not see that? Perspectives of nonconsumer mental health researchers on the benefits of collaborative research with consumers. *International journal of mental health nursing*, 27(4), 1230-1239. - Happell, B., Bennetts, W., Tohotoa, J., Platania-Phung, C., & Wynaden, D. (2016). Nothing without vision! The views of consumers and mental health nurses about consumer involvement in mental health nursing education. *Collegian*, 23(2), 241-248. - Horgan, A., Manning, F., Bocking, J., Happell, B., Lahti, M., Doody, R., ... & O'Donovan, M. (2018). ‘To be treated as a human’: Using co‐production to explore experts by experience involvement in mental health nursing education–The COMMUNE project. *International journal of mental health nursing*, 27(4), 1282-1291. |
| 1 | PLATANIA PHUNG C. | University of Newcastle, Australia | Australia |  |
| 1 | SCHOLZ B. | The Australian National University, Australia | Australia |  |
| 1 | BOCKING J. | University of Canberra, Australia | Australia |  |
| 1 | GORDON S. | University of Otago, New Zealand | New Zealand |  |
| 1 | ELLIS P. | University of Otago, New Zealand | New Zealand |  |
| 1 | ROPER C. | University of Melbourne, Australia | Australia |  |
| 1 | LIGGINS J. | University of Auckland, New Zealand | New Zealand |  |
| 1 | HORGAN A. | University College Cork, Ireland | Ireland |  |
| 1 | MANNING F. | University College Cork, Ireland | Ireland |  |
| 1 | DOODY R. | University College Cork, Ireland | Ireland |  |
| 1 | HALS E. | University of Applied Sciences, Norway | Norway |  |
| 1 | GRANERUD A. | University of Applied Sciences, Norway | Norway |  |
| 1 | LAHTI M. | Turku University, Finland | Finland |  |
| 1 | VAN DER VAART K.J. | University of Applied Sciences Utrecht, The Netherlands | The Netherlands |  |
| 1 | ALLON J. | University of Applied Sciences Utrecht, The Netherlands | The Netherlands |  |
| 1 | GRIFFIN M. | Dublin City University, Ireland | Ireland |  |
| 1 | RUSSELL S. | Dublin City University, Ireland | Ireland |  |
| 1 | MACGABHANN L. | Dublin City University, Ireland | Ireland |  |
| 1 | BJORNSSON E. | University of Iceland, Iceland | Iceland |  |
| 1 | BIERING P. | University of Iceland, Iceland | Iceland |  |
| 2 | HAMALAINEN R.M. | National Institute for Health and Welfare, Finland | Finland | - van de Goor, I., Hämäläinen, R. M., Syed, A., Lau, C. J., Sandu, P., Spitters, H., ... & Aro, A. R. (2017). Determinants of evidence use in public health policy making: Results from a study across six EU countries. *Health Policy*, 121(3), 273-281. - Hämäläinen, R. M., Aro, A. R., Lau, C. J., Rus, D., Cori, L., & Syed, A. M. (2016). Cross-sector cooperation in health-enhancing physical activity policymaking: more potential than achievements?. *Health research policy and systems*, 14(1), 33. - Tudisca, V., Valente, A., Castellani, T., Stahl, T., Sandu, P., Dulf, D., ... & Loncarevic, N. (2018). Development of measurable indicators to enhance public health evidence-informed policy-making*. Health research policy and systems*, 16(1), 47. |
| 2 | ARO A. | University of Southern Denmark, Denmark | Denmark |  |
| 2 | LAU C.J. | Research Centre for Prevention and Health, Denmark | Denmark |  |
| 2 | VALENTE A. | The National Research Council of Italy (CNR), Italy | Italy |  |
| 2 | CASTELLANI T. | The National Research Council of Italy (CNR), Italy | Italy |  |
| 2 | SANDU P. | Babeș-Bolyai University (BBU), Romania | Romania |  |
| 2 | DULF D. | Babeș-Bolyai University (BBU), Romania | Romania |  |
| 2 | SPITTERS H. | Tilburg University, The Netherlands | The Netherlands |  |
| 2 | VAN DE GOOR I. | Tilburg University, The Netherlands | The Netherlands |  |
| 3 | GREENHALGH T. | University of Oxford, UK | UK | - Procter, R., Wherton, J., & Greenhalgh, T. (2018). Hidden Work and the Challenges of Scalability and Sustainability in Ambulatory Assisted Living. *ACM Transactions on Computer-Human Interaction* (TOCHI), 25(2), 11. - Procter, R., Wherton, J., Greenhalgh, T., Sugarhood, P., Rouncefield, M., & Hinder, S. (2016). Telecare call centre work and ageing in place. Computer Supported Cooperative Work (CSCW), 25(1), 79-105; - Procter, R., Greenhalgh, T., Wherton, J., Sugarhood, P., Rouncefield, M., & Hinder, S. (2014). The day-to-day co-production of ageing in place. *Computer Supported Cooperative Work* (CSCW), 23(3), 245-267. - Wherton, J., Sugarhood, P., Procter, R., Hinder, S., & Greenhalgh, T. (2015). Co-production in practice: how people with assisted living needs can help design and evolve technologies and services. *Implementation Science*, 10(1), 75. - Wherton, J., Sugarhood, P., Procter, R., Rouncefield, M., Dewsbury, G., Hinder, S., & Greenhalgh, T. (2012). Designing assisted living technologies ‘in the wild’: preliminary experiences with cultural probe methodology. *BMC medical research methodology*, 12(1), 188. |
| 3 | PROCTER R. | Warwick University, UK | UK |  |
| 3 | WHERTON J. | Queen Mary University, UK | UK |  |
| 3 | SUGARHOOD P. | Newham University Hospital, UK | UK |  |
| 3 | ROUNCEFIELD M. | Lancaster University, UK | UK |  |
| 3 | HINDER S. | Queen Mary University, UK | UK |  |
| 4 | JENNINGS H. | York St. John University, UK | UK | - Jennings, H., Slade, M., Bates, P., Munday, E., & Toney, R. (2018). Best practice framework for Patient and Public Involvement (PPI) in collaborative data analysis of qualitative mental health research: methodology development and refinement. *BMC psychiatry*, 18(1), 213. - Toney, R., Elton, D., Munday, E., Hamill, K., Crowther, A., Meddings, S., ... & Pollock, K. (2018). Mechanisms of action and outcomes for students in Recovery Colleges*. Psychiatric Services*, 69(12), 1222-1229. |
| 4 | SLADE M. | University of Nottingham, UK | UK |  |
| 4 | BATES P. | Peter Bates Associates Ltd, UK | UK |  |
| 4 | MUNDAY E. | RECOLLECT Lived Experience Advisory Panel, UK | UK |  |
| 4 | TONEY R. | University of Nottingham, UK | UK |  |
| 5 | FREEBAIRN L. | ACT Health / Sax Institute/University of Notre Dame, Australia | Australia | - Freebairn, L., Atkinson, J. A., Kelly, P. M., McDonnell, G., & Rychetnik, L. (2018). Decision makers’ experience of participatory dynamic simulation modelling: methods for public health policy. *BMC medical informatics and decision making*, 18(1), 131. - Freebairn, L., Rychetnik, L., Atkinson, J. A., Kelly, P., McDonnell, G., Roberts, N., ... & Redman, S. (2017). Knowledge mobilisation for policy development: implementing systems approaches through participatory dynamic simulation modelling. *Health research policy and systems*, 15(1), 83. |
| 5 | ATKINSON J.A. | Sax Institute/ University of Sydney, Australia | Australia |  |
| 5 | MCDONNELL G. | Sax Institute, Australia | Australia |  |
| 5 | RYCHETNIK L. | Sax Institute/University of Notre Dame, Australia | Australia |  |
| 5 | KELLY P. | ACT Health / Sax Institute/The Australian National University, Australia | Australia |  |
| 5 | REDMAN S. | Sax Institute, Australia | Australia |  |
| 6 | MOORE L. | University of Glasgow, UK | UK | - Hawkins, J., Madden, K., Fletcher, A., Midgley, L., Grant, A., Cox, G., Moore, L., R.Campbell, R., Murphy, S.,Bonell, C.,& White, J. (2017). Development of a framework for the co-production and prototyping of public health interventions. *BMC public health*, 17(1), 689. - Williams, A., Moore, S. C., Shovelton, C., Moore, L., & Murphy, S. (2016). Process evaluation of an environmental health risk audit and action plan intervention to reduce alcohol related violence in licensed premises. *BMC public health*, 16(1), 455. |
| 6 | MURPHY S. | Cardiff University, UK | UK |  |
| 7 | KISLOV R | University of Manchester/ CLAHRCs, UK | UK | - Harvey, G., Fitzgerald, L., Fielden, S., McBride, A., Waterman, H., Bamford, D., ... & Boaden, R. (2011). The NIHR collaboration for leadership in applied health research and care (CLAHRC) for Greater Manchester: combining empirical, theoretical and experiential evidence to design and evaluate a large-scale implementation strategy. *Implementation Science*, 6(1), 96. - Kislov, R., Wilson, P. M., Knowles, S., & Boaden, R. (2018). Learning from the emergence of NIHR Collaborations for Leadership in Applied Health Research and Care (CLAHRCs): a systematic review of evaluations. *Implementation Science*, 13(1), 111. |
| 7 | BOADEN R | University of Manchester/ CLAHRCs, UK | UK |  |
| 8 | SANDERS C. | University of Manchester, UK | UK | - Knowles, S., Hays, R., Senra, H., Bower, P., Locock, L., Protheroe, J., ... & Daker‐White, G. (2018). Empowering people to help speak up about safety in primary care: Using codesign to involve patients and professionals in developing new interventions for patients with multimorbidity. *Health Expectations*, 21(2), 539-548. - Rhodes, P., McDonald, R., Campbell, S., Daker‐White, G., & Sanders, C. (2016). Sensemaking and the co‐production of safety: a qualitative study of primary medical care patients. *Sociology of health & illness*, 38(2), 270-285. |
| 8 | DAKER-WHITE G. | University of Manchester, UK | UK |  |
| 9 | BUNN F. | University of Hertfordshire, UK | UK | - Bunn, F., Goodman, C., Manthorpe, J., Durand, M. A., Hodkinson, I., Rait, G., ... & Wilson, P. (2017). Supporting shared decision-making for older people with multiple health and social care needs: a protocol for a realist synthesis to inform integrated care models. *BMJ open*, 7(2), e014026. - Bunn, F., Burn, A. M., Robinson, L., Poole, M., Rait, G., Brayne, C., ... & Goodman, C. (2017). Healthcare organisation and delivery for people with dementia and comorbidity: a qualitative study exploring the views of patients, carers and professionals. *BMJ open*, 7(1), e013067.; |
| 9 | GOODMAN C. | University of Hertfordshire, UK | UK |  |
| 9 | RAIT G. | University College London, UK | UK |  |
| 10 | BROPHY L | University of Melbourne, Australia | Australia | - Petrakis, M., Brophy, L., Lewis, J., Stylianou, M., Scott, M., Cocks, N., ... & Halloran, K. (2014). Consumer measures and research co-production: A pilot study evaluating the recovery orientation of a mental health program collaboration. *Asia Pacific Journal of Social Work and Development,* 24(1-2), 94-108. - Brophy, L., Bruxner, A., Wilson, E., Cocks, N., & Stylianou, M. (2015). How social work can contribute in the shift to personalised, recovery-oriented psycho-social disability support services. *British Journal of Social Work*, 45(suppl_1), i98-i116. |
| 10 | COCKS N | Mind Australia, Australia | Australia |  |
| 10 | STYLIANOU M | Mind Australia, Australia | Australia |  |
| 11 | FARMER J. | Swinburne University, Australia | Australia | - Farmer, J., Carlisle, K., Dickson-Swift, V., Teasdale, S., Kenny, A., Taylor, J., ... & Gussy, M. (2018). Applying social innovation theory to examine how community co-designed health services develop: using a case study approach and mixed methods. *BMC health services research*, 18(1), 68 - Farmer, J., Currie, M., Kenny, A., & Munoz, S. A. (2015). An exploration of the longer-term impacts of community participation in rural health services design. *Social Science & Medicine*, 141, 64-71. - Farmer, - J., Taylor, J., Stewart, E., & Kenny, A. (2018). Citizen participation in health services co-production: a roadmap for navigating participation types and outcomes. *Australian journal of primary health*, 23(6), 509-515. |
| 11 | KENNY A. | La Trobe University, Australia | Australia |  |
| 11 | TAYLOR J. | James Cook University, Australia | Australia |  |
| 12 | LATIF A. | University of Nottingham, UK | UK | - Latif, A., Carter, T., Rychwalska-Brown, L., Wharrad, H., & Manning, J. (2017). Co-producing a digital educational programme for registered children’s nurses to improve care of children and young people admitted with self-harm. *Journal of child health care*, 21(2), 191-200. - Latif, A., Pollock, K., Anderson, C., Waring, J., Solomon, J., Chen, L. C., ... & Wharrad, H. (2016). Supporting underserved patients with their medicines: a study protocol for a patient/professional coproduced education intervention for community pharmacy staff to improve the provision and delivery of Medicine Use Reviews (MURs). *BMJ open*, 6(12), e013500. - Toney, R., Elton, D., Munday, E., Hamill, K., Crowther, A., Meddings, S., ... & Pollock, K. (2018). Mechanisms of action and outcomes for students in Recovery Colleges*. Psychiatric Services*, 69(12), 1222-1229. |
| 12 | WHARRAD H. | University of Nottingham, UK | UK |  |
| 12 | POLLOCK K. | University of Nottingham, UK | UK |  |
| 12 | WARING J. | University of Nottingham, UK | UK |  |
