# Supplementary material for: Co-production in health policy and management: a comprehensive bibliometric review
Source: BMC Health Serv Res. 2020 Jun 5;20:504. doi: 10.1186/s12913-020-05241-2 (PMC7275357; doi:10.1186/s12913-020-05241-2)
Supplement: Supplementary file 5 — Additional file 5. List of intellectual structure clusters [file 12913_2020_5241_MOESM5_ESM.docx]

***Additional file 5: List of references in co-citation analysis***

| **Node** | **Group** | **Cluster** | **Complete Reference** |
| --- | --- | --- | --- |
| BOVAIRD T 2007-1 | A | 1 | Bovaird, T. (2007). Beyond engagement and participation: User and community coproduction of public services. Public administration review, 67(5), 846-860 |
| OSBORNE SP 2013-1 | A | 1 | Osborne, S. P., & Strokosch, K. (2013). It takes Two to Tango? Understanding the Co‐production of Public Services by Integrating the Services Management and Public Administration Perspectives. British Journal of Management, 24, S31-S47. |
| OSBORNE SP 2013-2 | A | 1 | Osborne, S. P., Radnor, Z., & Nasi, G. (2013). A new theory for public service management? Toward a (public) service-dominant approach. The American Review of Public Administration, 43(2), 135-158. |
| VERSCHUERE B 2012 | A | 1 | Verschuere, B., Brandsen, T., & Pestoff, V. (2012). Co-production: The state of the art in research and the future agenda. Voluntas: International Journal of Voluntary and Nonprofit Organizations, 23(4), 1083-1101. |
| DUNSTON R 2009 | A | 1 | Dunston, R., Lee, A., Boud, D., Brodie, P., & Chiarella, M. (2009). Co‐production and health system reform–from re‐imagining to re‐making. Australian Journal of Public Administration, 68(1), 39-52 |
| ALFORD J 2009-1 | A | 1 | Alford, J. (2009). Engaging public sector clients: from service-delivery to co-production. Springer |
| HARDYMAN W 2015 | A | 1 | Hardyman, W., Daunt, K. L., & Kitchener, M. (2015). Value co-creation through patient engagement in health care: a micro-level approach and research agenda. Public Management Review, 17(1), 90-107. |
| PESTOFF V 2006 | A | 1 | Pestoff, V. (2006). Citizens and co-production of welfare services: Childcare in eight European countries. Public management review, 8(4), 503-519. |
| BRUDNEY JL 1983-1 | A | 1 | Brudney, J. L., & England, R. E. (1983). Toward a definition of the coproduction concept. Public administration review, 59-65. |
| PARKS RB 1981 | A | 1 | Parks, R. B., Baker, P. C., Kiser, L., Oakerson, R., Ostrom, E., Ostrom, V., ... & Wilson, R. (1981). Consumers as coproducers of public services: Some economic and institutional considerations. Policy Studies Journal, 9(7), 1001-1011. |
| BRANDSEN T 2006 | A | 1 | Brandsen, T., & Pestoff, V. (2006). Co-production, the third sector and the delivery of public services: An introduction. Public management review, 8(4), 493-501. |
| ALFORD J 2002-1 | A | 1 | Alford, J. (2002). Why do public-sector clients coproduce? Toward a contingency theory. Administration & Society, 34(1), 32-56. |
| WHITAKER GP 1980 | A | 1 | Whitaker, G. P. (1980). Coproduction: Citizen participation in service delivery. Public administration review, 240-246. |
| RADNOR Z 2014 | A | 1 | Radnor, Z., Osborne, S. P., Kinder, T., & Mutton, J. (2014). Operationalizing co-production in public services delivery: The contribution of service blueprinting. Public Management Review, 16(3), 402-423. |
| REALPE A 2010-1 | A | 1 | Realpe, A., & Wallace, L. M. (2010). What is co-production. London: The Health Foundation, 1-11. |
| ALFORD J 2014 | A | 1 | Alford, J. (2014). The multiple facets of co-production: Building on the work of Elinor Ostrom. Public Management Review, 16(3), 299-316. |
| FOTAKI M 2011 | A | 1 | Fotaki, M. (2011). Towards developing new partnerships in public services: Users as consumers, citizens and/or co‐producers in health and social care in England and Sweden. Public administration, 89(3), 933-955. |
| NEEDHAM C 2008-1 | A | 1 | Needham, C. (2008). Realising the potential of co-production: negotiating improvements in public services. *Social policy and society*, *7*(2), 221-231. |
| BOVAIRD T. 2012 | A | 1 | Bovaird, T., & Loeffler, E. (2012). From engagement to co-production: The contribution of users and communities to outcomes and public value. Voluntas: International Journal of Voluntary and Nonprofit Organizations, 23(4), 1119-1138. |
| VAN EIJK 2014 | A | 1 | Van Eijk, C. J., & Steen, T. P. (2014). Why people co-produce: Analysing citizens’ perceptions on co-planning engagement in health care services. Public Management Review, 16(3), 358-382. |
| FLEDDERUS J 2014-2 | A | 1 | Fledderus, J., Brandsen, T., & Honingh, M. (2014). Restoring trust through the co-production of public services: A theoretical elaboration. Public Management Review, 16(3), 424-443 |
| EISENHARDT KM 1989 | A | 1 | Eisenhart, K. M. (1989). Building theories from case study research. Academy of management review, 14(4), 532-550. |
| PESTOFF V. 2012-1 | A | 1 | Pestoff, V. 2012. ‘New Public Governance, CoProduction and Third Sector Social Services in Europe: Crowding In and Crowding Out’. In V. Pestoff, T. Brandsen and B. Verschuere (eds.), New Public Governance, the Third Sector and Co-Production (pp. 361–380). New York: Routledge |
| OSTROM E 1996 | A | 2 | Ostrom, E. (1996). Crossing the great divide: coproduction, synergy, and development. World development, 24(6), 1073-1087. |
| VOORBERG WH 2015 | A | 2 | Voorberg, W. H., Bekkers, V. J., & Tummers, L. G. (2015). A systematic review of co-creation and co-production: Embarking on the social innovation journey. Public Management Review, 17(9), 1333-1357. |
| BOYLE D. 2009 | A | 2 | Boyle, D., & Harris, M. (2009). The challenge of co-production. London: New Economics Foundation. |
| BATALDEN M 2016 | A | 2 | Batalden, M., Batalden, P., Margolis, P., Seid, M., Armstrong, G., Opipari-Arrigan, L., & Hartung, H. (2016). Coproduction of healthcare service. BMJ Quality & Safety, 25, 509-517. |
| NEEDHAM C. 2009 | A | 2 | Needham, C., & Carr, S. (2009). SCIE Research Briefing 31: Co-production: An emerging evidence base for adult social care transformation. Policing, 8(11). |
| OSBORNE SP 2016 | A | 2 | Osborne, S. P., Radnor, Z., & Strokosch, K. (2016). Co-production and the co-creation of value in public services: a suitable case for treatment?. Public Management Review, 18(5), 639-653. |
| SLAY J 2013 | A | 2 | Slay, J., & Stephens, L. (2013). Co-production in mental health: A literature review. London: new economics foundation. |
| JASANOFF S 2004 | A | 2 | Jasanoff, S. (Ed.). (2004). States of knowledge: the co-production of science and the social order. Routledge. |
| BRAUN V 2006 | A | 3 | Braun, V., & Clarke, V. (2006). Using thematic analysis in psychology. Qualitative research in psychology, 3(2), 77-101. |
| BRETT J 2014-1 | A | 3 | Brett, J., Staniszewska, S., Mockford, C., Herron‐Marx, S., Hughes, J., Tysall, C., & Suleman, R. (2014). Mapping the impact of patient and public involvement on health and social care research: a systematic review. Health Expectations, 17(5), 637-650. |
| VARGO SL 2008-2 | B | 4 | Vargo, S. L., & Lusch, R. F. (2008). Service-dominant logic: continuing the evolution. Journal of the Academy of marketing Science, 36(1), 1-10. |
| VARGO STEPHEN 2008 | B | 4 | Vargo, S. L., Maglio, P. P., & Akaka, M. A. (2008). On value and value co-creation: A service systems and service logic perspective. *European management journal*, 26(3), 145-152. |
| BENDAPUDI N 2003 | B | 4 | Bendapudi, N., & Leone, R. P. (2003). Psychological implications of customer participation in co-production. Journal of marketing, 67(1), 14-28. |
| LUSCH RF 2006 | B | 4 | Lusch, R. F., & Vargo, S. L. (2006). Service-dominant logic: reactions, reflections and refinements. Marketing theory, 6(3), 281-288. |
| VARGO SL 2004 | B | 4 | Vargo, S. L., & Lusch, R. F. (2004). Evolving to a new dominant logic for marketing. Journal of marketing, 68(1), 1-17 |
| BERRY LL 2007 | B | 4 | Berry, L. L., & Bendapudi, N. (2007). Health care: a fertile field for service research. Journal of Service Research, 10(2), 111-122. |
| MCCOLL-KENNEDY JR 2012 | B | 4 | McColl-Kennedy, J. R., Vargo, S. L., Dagger, T. S., Sweeney, J. C., & Kasteren, Y. V. (2012). Health care customer value cocreation practice styles. Journal of Service Research, 15(4), 370-389. |
| DELLANDE S 2004 | B | 4 | Dellande, S., Gilly, M. C., & Graham, J. L. (2004). Gaining compliance and losing weight: the role of the service provider in health care services. Journal of marketing, 68(3), 78-91. |
| GAGLIARDI AR 2016-1 | C | 5 | Gagliardi, A. R., Berta, W., Kothari, A., Boyko, J., & Urquhart, R. (2015). Integrated knowledge translation (IKT) in health care: a scoping review. Implementation Science, 11(1), 38. |
| GRAHAM ID 2006 | C | 5 | Graham, I. D., Logan, J., Harrison, M. B., Straus, S. E., Tetroe, J., Caswell, W., & Robinson, N. (2006). Lost in knowledge translation: time for a map?. Journal of continuing education in the health professions, 26(1), 13-24. |
| GREENHALGH T 2016-1 | C | 5 | Greenhalgh, T., Jackson, C., Shaw, S., & Janamian, T. (2016). Achieving research impact through co‐creation in community‐based health services: literature review and case study. The Milbank Quarterly, 94(2), 392-429. |
| RYCROFT-MALONE J 2011-1 | C | 5 | Rycroft-Malone, J., Wilkinson, J. E., Burton, C. R., Andrews, G., Ariss, S., Baker, R., ... & McCormack, B. G. (2011). Implementing health research through academic and clinical partnerships: a realistic evaluation of the Collaborations for Leadership in Applied Health Research and Care (CLAHRC). Implementation Science, 6(1), 74. |
| KOTHARI A 2011 | C | 5 | Kothari, A., MacLean, L., Edwards, N., & Hobbs, A. (2011). Indicators at the interface: managing policymaker-researcher collaboration. Knowledge Management Research & Practice, 9(3), 203-214. |
| OLIVER K 2014-1 | C | 5 | Oliver, K., Innvar, S., Lorenc, T., Woodman, J., & Thomas, J. (2014). A systematic review of barriers to and facilitators of the use of evidence by policymakers. BMC health services research, 14(1), 2. |
| NUTLEY S. 2007 | C | 5 | Nutley, S. M., Walter, I., & Davies, H. T. (2007). Using evidence: How research can inform public services. Policy press. |
| GIBBONS M 1994 | C | 5 | Gibbons, M. (Ed.). (1994). The new production of knowledge: The dynamics of science and research in contemporary societies. Sage. |
| OLIVER K 2014-2 | C | 5 | Oliver, K., Lorenc, T., & Innvær, S. (2014). New directions in evidence-based policy research: a critical analysis of the literature. Health research policy and systems, 12(1), 34. |
